# Semantic learning from keyframe demonstration using object attribute constraints

**DOI:** 10.3389/frobt.2024.1340334

**Published:** 2024-07-18

**Authors:** Busra Sen, Jos Elfring, Elena Torta, René van de Molengraft

**Affiliations:** Department of Mechanical Engineering, Eindhoven University of Technology, Eindhoven, Netherlands

**Keywords:** learning from demonstration, keyframe demonstrations, object attributes, task goal learning, semantic learning

## Abstract

Learning from demonstration is an approach that allows users to personalize a robot’s tasks. While demonstrations often focus on conveying the robot’s motion or task plans, they can also communicate user intentions through object attributes in manipulation tasks. For instance, users might want to teach a robot to sort fruits and vegetables into separate boxes or to place cups next to plates of matching colors. This paper introduces a novel method that enables robots to learn the semantics of user demonstrations, with a particular emphasis on the relationships between object attributes. In our approach, users demonstrate essential task steps by manually guiding the robot through the necessary sequence of poses. We reduce the amount of data by utilizing only robot poses instead of trajectories, allowing us to focus on the task’s goals, specifically the objects related to these goals. At each step, known as a keyframe, we record the end-effector pose, object poses, and object attributes. However, the number of keyframes saved in each demonstration can vary due to the user’s decisions. This variability in each demonstration can lead to inconsistencies in the significance of keyframes, complicating keyframe alignment to generalize the robot’s motion and the user’s intention. Our method addresses this issue by focusing on teaching the higher-level goals of the task using only the required keyframes and relevant objects. It aims to teach the rationale behind object selection for a task and generalize this reasoning to environments with previously unseen objects. We validate our proposed method by conducting three manipulation tasks aiming at different object attribute constraints. In the reproduction phase, we demonstrate that even when the robot encounters previously unseen objects, it can generalize the user’s intention and execute the task.

## 1 Introduction

As robots become increasingly integrated into diverse environments, from factories to homes, the need for more intuitive and adaptable programming methods becomes paramount. Traditional robot programming methods fall into two main categories: online and offline programming (Pan et al., 2012). Online programming includes methods like lead-through, where the user records the robot’s path via a teach-pendant, and walk-through, where the user physically guides the robot through the desired motions (Pan et al., 2012; Villani et al., 2018). This approach is user-friendly as it doesn’t require programming skills. However, its lack of adaptability necessitates the re-recording of the entire sequence even for minor changes in the environment (Villani et al., 2018). Offline programming, on the other hand, involves defining each robot’s movement for a specific task (Pan et al., 2012). This approach is often limited to highly controlled environments and requires expertise in robotics.

Inspired by people’s ability to learn by imitating others (Calinon, 2018), Learning from Demonstration (LfD) offers a promising solution to these limitations. Unlike offline robot programming, LfD does not require explicit programming of a robot’s task. Instead, it allows users to teach robots their skills through demonstrations. LfD does not merely repeat pre-recorded robot motions like online robot programming (Ravichandar et al., 2020). In each demonstration, LfD approaches can extract the task constraints implicitly and generalize these learned skills to different environments (Hussein et al., 2018; Ravichandar et al., 2020). Moreover, it can handle a wider range of tasks compared to traditional offline robot programming. This is evidenced by its successful applications in various fields such as healthcare tasks (Fong et al., 2019; Pareek and Kesavadas, 2020), household tasks (Ye and Alterovitz, 2017), and industrial tasks (Ramirez-Amaro et al., 2019).

The process of LfD begins with a demonstration phase, where the user imparts relevant information about the robot’s state and the environment through demonstrations. These demonstrations can be performed through kinesthetic teaching, teleoperation, or passive observation (Ravichandar et al., 2020). Following this, the robot learns to encode the recorded data to identify commonalities among the demonstrations. The encoding method typically depends on the learning paradigm, which could be at a low level (e.g., robot trajectories) or a high level (e.g., action order, semantic rules) (Billard et al., 2008).

At low-level LfD, the demonstrated trajectories, which are continuous sequences of waypoints with timestamps, can be parameterized in either the joint and/or task space (Chernova and Thomaz, 2014). To generalize these trajectories to different initial robot states or different object positions, the user needs to provide multiple demonstrations, which can increase their workload (Akgun et al., 2012b). Moreover, since trajectories include time information, the quality of the user’s demonstration is crucial to avoid jerky movements. Recent studies have proposed learning from time-independent sparse data, known as keyframes, instead of recording full trajectories at a high rate (Akgun et al., 2012b). These keyframe or trajectory demonstrations typically include robot states (i.e., joint angles, end-effector poses) and information about the poses of objects involved in the task. However, to semantically understand the goal behind a demonstration, more than just a robot pose or object pose is needed.

**FIGURE 1:**
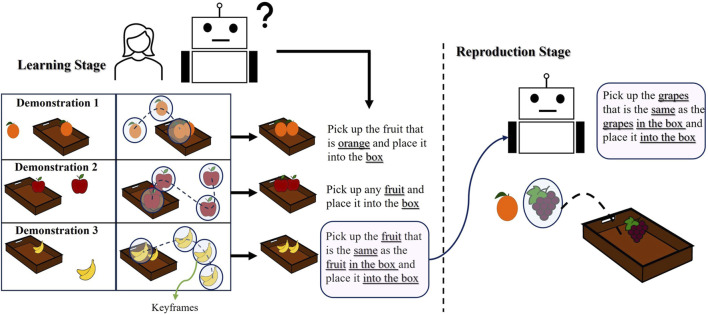
The user changes the objects used in the demonstrations to teach her intention. Our proposed method computes similarities between demonstrations and within a demonstration from keyframes. The robot learns the purpose of the task from the object attribute relations.

Understanding the user’s intent about the task goals or which objects the robot should give attention to are part of issues that need to be addressed when adopting the high-level LfD approaches (Fonooni et al., 2015). In this study, the term “user intention” denotes any task goals related to the objects used throughout the task, and the attributes of the objects (such as color, shape, size, etc.) that are relevant for understanding the task goals. To generalize task goals regarding object attributes, the user is expected to change the objects in each demonstration to meet her/his intentions. For example, in Figure 1, the user wants to collect the same fruits in a box. If the user only demonstrates the task once, the robot might not learn if the task is about the specific type of fruit used in the demonstration or about fruit in general. However, demonstrating the task multiple times with different fruits allows for generalization.

In the first demonstration, the robot learns to “pick up the orange fruits and place it into the box.” In the second demonstration, the user changes the objects, and then the robot learns to “pick up any fruit and place it into the box”. In the last demonstration, the robot learns that “the fruit in the box must be the same as the fruit to be picked”. After three demonstrations, the robot learns to “pick up the fruit that is the same as the fruit in the box and place it into the box”. During task reproduction, the robot may encounter different fruits, but it should know the desired constraints (the fruit in the box and the fruit to be picked must be the same). The robot does not need to see these objects during teaching. It checks the fruit in the box and then finds the fruit that satisfies the constraint. Giving attention to the objects allows the robot to generalize the user’s intention to unseen environments by uncovering a set of similar objects’ attributes used in each demonstration. These task goals, or user intentions, are taught implicitly; the only thing the user needs to do is to change the objects to convey her intention and bring the robot to the desired robot poses during demonstrations to fulfil the intended task. The end-effector pose, object poses, and object attributes are recorded as a keyframe at each step.

The distribution of these keyframes depends on the task’s requirements and user decisions, so we cannot assume users will use the same number of keyframes for different demonstrations meant to teach the same task. Even among multiple demonstrations of the same task, the number of keyframes can differ. For instance, in Figure 1, the user records three keyframes in the first demonstration, four in the second, and four in the last demonstration. For an adequate summary of a task, a minimum number of keyframes is required for the robot to understand the task, as explained later. Non-expert users can, however, be expected to record additional keyframes that contain superfluous information and complicate learning. An alignment method is required to cluster the keyframes from different demonstrations to generalize the robot’s motion. After alignment, clusters can be a mix of required and unrequired keyframes. Required keyframes, referred to as object-centric, direct attention to the objects that reflect the user’s intention and provide accurate relative poses between the end-effector and the objects. Object-centric keyframes are defined as those that change an object’s attribute in the environment. Task-unimportant keyframes, on the other hand, are defined as remaining keyframes that do not alter any attribute during the demonstrations. We consider that novice users might record these task-unimportant keyframes. In summary, our research is motivated by the following challenges:

➢ The learning becomes more complicated when there is a varying number of keyframes. How can we extract conceptually duplicate keyframes from each demonstration?
➢ How can we derive the user’s intention regarding object attributes to generalize the task to previously unseen scenes?

This paper is organized as follows: Section 2 summarizes the related work on keyframes and task goals concerning the objects’ relationships in learning from demonstration, and it outlines our contributions to this field. Section 3 explains our proposed solution. Section 4 presents the experimental setup, and Section 5 validates our results by demonstrating various tasks that include different object attributes. Finally, Section 6 discusses the results, highlights the current limitations, and suggests possible future research directions.

## 2 Related work

Trajectory demonstrations are commonly used in literature to show robot motions. These demonstrations are instrumental in scenarios where velocity and timing are crucial to the required skills. However, due to their uninterrupted nature, demonstrations by novice users operating high-degree-of-freedom robots can be challenging (Chernova and Thomaz, 2014). Furthermore, multiple demonstrations are necessary to generalize the demonstrated skills to task parameters such as different object poses and varying initial robot positions. As the duration of each demonstration may differ, a time alignment method is employed to align these trajectories (Muhlig et al., 2009). It is also important to note that these trajectories’ smoothness depends on the user’s abilities. Therefore, optimization techniques are often required to prevent jerky movements (Ravichandar et al., 2020).

Akgun et al. (2012b) proposed using keyframe demonstrations to circumvent the issues above. Keyframes are described as sparse robot poses in joint configuration or task space. These keyframes are similar to the sub-goals that are highlighted after trajectory segmentations to learn task plans similar to the approach proposed by Lioutikov et al. (2015) and Niekum et al. (2015). However, in keyframe demonstrations, these sparse poses are given by the user. Experiments conducted by Akgun et al. (2012b) where novice users demonstrate keyframes, highlight the advantages of keyframes over trajectories. While these studies by Akgun et al. (2012b) do not primarily focus on generalization capabilities, multiple demonstrations would be more straightforward with keyframes as they do not present time alignment issues.

Despite these advantages, learning robot skills with keyframes is rare. One disadvantage is the lack of time information. In the field of robot motion generation from keyframes, various methods have been proposed to address this problem. Jankowski et al. (2022) developed a technique that creates smooth and adaptable robot trajectories from sparse key position demonstrations. This method solves a time-optimal control problem for each key position and adapts in real time to the current state of the robot and the environment. On the other hand, Akgun et al. (2012b) proposed a method that generates a sparse trajectory of joint angles, automatically adds keyframes for start and/or end positions if they are omitted, and calculates time data for each keyframe based on distance and a constant average velocity.

An alignment and/or a clustering method are required to extract similar keyframes/trajectories from multiple demonstrations. The approach by Akgun et al. (2012a) involves temporally aligning multiple skill demonstrations using an iterative process. This process employs Dynamic Time Warping (DTW) and an alignment pool, and sequences are selected based on the lowest pairwise DTW cost. Keyframes aligned to the same keyframe from another demonstration are clustered together. The maximum number of keyframes is chosen as the cluster number, and the Gaussian Mixture Model (GMM) is applied to cluster the keyframes (Akgun et al., 2012b). In the studies by Kurenkov et al. (2015); Perez-D’Arpino and Shah (2017) the number of clusters is determined by the rounded average number of keyframes, and K-means is used for clustering. Another approach by Akgun and Thomaz, (2016) uses Hidden Markov Models, initializing the number of states as the minimum number of keyframes. This learning model is applied separately for goal keyframes and action keyframes. Lastly, the study by Jankowski et al. (2022) operates under the assumption that the number of keyframes is identical among demonstrations. In these previous studies, to generalize the robot motion, either the number of keyframes is assumed to be constant, or alignment or clustering methods are used to deal with a varying number of keyframes. We remove the assumption of an equal number of keyframes in each demonstration, but also we do not use alignment or clustering methods to remove the dependency of generalization capabilities to these methods.

Although Akgun and Thomaz (2016) record the objects’ states as a goal keyframe to monitor the execution of action keyframes, they assume that there exists one object in the task. Differently, in our study, we do not assume which objects are important for the task, so we record the end-effector pose, all object attributes in the environment, and their poses as a keyframe. This definition provides the extraction of object-centric keyframes, even if the user records both object-centric keyframes and task-unimportant keyframes. These object-centric keyframes help us to give attention to the objects and guarantee that relative poses between the end-effector and objects would not be affected by incorrect clustering of keyframes because of task-unimportant keyframes.

We focus on understanding user intent behind demonstrations by observing objects. Inverse Reinforcement Learning (IRL) also aims to comprehend user intent by recovering a reward function that justifies expert demonstrations (Ab Azar et al., 2020; Arora and Doshi, 2021). This function is often estimated from expert trajectories in robotics for various applications, such as navigation for socially compliant robots (Kretzschmar et al., 2016; Kollmitz et al., 2020; Sun et al., 2020) and path and velocity preferences for robot arms (Bobu et al., 2018; Avaei et al., 2023). IRL typically constrains the reward space with predefined features (Shek et al., 2023), except for some recent work that updates these features online (Lourenço et al., 2023). These features usually include relative distances or velocity and acceleration preferences. Unlike IRL, we don’t learn a reward function but have a predefined object attribute set. Our study’s contribution is teaching semantics about these attributes from keyframe demonstrations and generalizing this to unseen environments.

In robotics, semantics can be viewed as a robot’s ability to understand the significance of environmental entities. Numerous applications have been developed incorporating semantics. For example, mobile robots construct semantic maps (Deng et al., 2024) enhancing navigation (Qi et al., 2020; Achat et al., 2023) and facilitating dynamic object searches in domestic settings (Guo et al., 2022; Zhang et al., 2023). Human-robot interaction (HRI) uses semantics to generate natural questions about environmental objects (Moon and Lee, 2020), execute tasks specified in natural language using Natural Language Processing (NLP) (Bucker et al., 2022; 2023), and physical human-robot interaction (Lourenço et al., 2023). In robot manipulation, semantics is used in object grasping based on attributes like fragility or softness (Kwak et al., 2022). These are a few examples of semantics applications in robotics. However, our paper focuses on a subset of applications related to the use of semantics for LfD in industrial robot manipulation tasks.

Semantics in LfD can be used to simplify user programming. Eiband et al. (2023) used position and force-torque sensing to identify semantic skills, which were classified using a support vector machine. A decision tree classifier was used to understand the correlation between the robot’s movements, environmental data, and activities (Ramirez-Amaro et al., 2019). Steinmetz et al. (2019) identified skills from a predefined set, described them using the Planning Domain Definition Language (PDDL), and conveyed semantically annotated skills to the user via an interface. Zanchettin (2023) proposed a method for semantically representing demonstrated skills, enabling the robot to identify workspace elements and understand the skill’s preconditions and effects.

One purpose of using object attributes in LfD is to select the appropriate actions; Chella et al. (2006) use conceptual spaces introduced by Peter (2000) which are metric spaces, formed by quality dimensions such as time, color, shape, and weight. The proposed approach by Chella et al. (2006) decides which action the robot should perform, based on the similarities between the scenes in the reproduction and demonstration actions. Differently, Fonooni et al. (2012) use semantic networks in LfD to generalize learned skills to new objects and situations by comparing network nodes before and after the demonstration. The environment in the demonstration and the environment in the reproduction can be different. Transfer learning and object mapping are applied in LfD to generalize the task to unseen environments (Bullard et al., 2016; Fitzgerald et al., 2018). Although these studies consider object attributes to generalize the demonstrations to unseen environments, they do not take into account the user intention related to object attributes.

Kaelbling et al. (2001) state that *“It is hard to imagine a truly intelligent agent that does not conceive of the world in terms of objects and their properties and relations to other objects.”* This statement is the main idea of our research, and its importance is also shown by the research of Cubek et al. (2015) which inspired our research. For example, spatial relations between objects might be crucial for the robot to define task goals (French et al., 2023) represent the task goal as spatial relations between scene objects, using a scene graph with objects as nodes and inter-object relations as edges. The aim is to generalize spatial relation goals to different environments, including those with varied objects. Similarly, spatial relations (distance and angle for labeled objects) between the served object and other table objects are learned for a food-serving task, enabling the robot to identify possible object arrangements in unseen scenes (Kawasaki and Takahashi, 2021). Hristov and Ramamoorthy (2021) label demonstrated trajectories using high-level concepts, including spatial labels like “behind” and “on top of,” and temporal labels like “quickly.” This approach helps in generating new trajectories by combining these labels.

The approach proposed by Chao et al. (2010) involves learning a set of criteria for an unchanged object’s attributes such as color to meet some expectations such as location. Fonooni et al. (2015) expanded their previous work by adding the Ant Colony Algorithm to determine the relevant nodes in the Semantic Network to focus on significant aspects of the demonstration, such as the shape of the object. Then they add priming to their method to reduce the number of demonstrations required in similar contexts (Fonooni et al., 2016). Ramirez-Amaro et al. (2019) proposed an approach using an ontology-based graph to adjust a demonstrated skill based on object attributes. For example, if a task is demonstrated using an orange, and the perceived object is an apple, the task steps are modified as there is no “squeeze” activity for the apple, without requiring a new demonstration. A recent study by Du et al. (2024) focused on sorting tasks based on the object colors, noting key trajectory positions to execute the task in various environments. However, their study only records and matches the pick and target objects’ colors, not considering the intention between demonstrations or object attribute relations. Our work’s goal is much closer to the research of Cubek et al. (2015). In their approach, they change the used objects in every demonstration to understand the user’s goal. They focus on pick-and-place tasks, and they extract similar object attributes for active (grasped) and passive (released) objects between demonstrations. However Chao et al. (2010) and Cubek et al. (2015), do not consider the possible similarities within demonstrations, Fonooni et al. (2015; 2016) assume that a semantic network includes all the necessary concepts and objects that the robot can work with, and nodes of the semantic network are discrete. Moreover, while in previous studies, tasks were handled as involving two objects, in this study, we define the relevant objects in the environment more comprehensively, and this allows us to teach more complex tasks. Our contributions can be summarized as follows:

• We propose a keyframe-based learning approach in which non-required keyframes are automatically identified and removed such that demonstrations with varying number of keyframes can be handled.
• The proposed method identifies similarities between discrete and continuous object attributes of objects within a single demonstration as well as across demonstrations, thereby allowing for a better generalization to unseen scenes and the ability to learn more complicated tasks.
• The proposed method has been validated through a comprehensive set of LfD experiments. Implicitly, user intentions regarding object attributes are extracted in three different tasks: stacking, sorting, and serving.
  ✓ In the stacking task, we demonstrate that discrete attributes of objects, such as color and shape, play an important role.
  ✓ The sorting task involves an increased number of objects, showcasing the proposed solution’s ability to handle both continuous (size) and discrete (shape) attributes successfully.
  ✓ Finally, in the serving task, we reveal that user desires may include objects observed in the environment throughout the task, even if they are not actively used by the robot.

These experiments highlight the method’s capacity to generalize tasks beyond those previously presented in related work.

To the authors’ knowledge, this is the first study that learns user intentions regarding the relations between the attributes of objects and generalizes this intention to unseen scenes by taking advantage of keyframe demonstrations.

## 3 Methods

**FIGURE 2:**
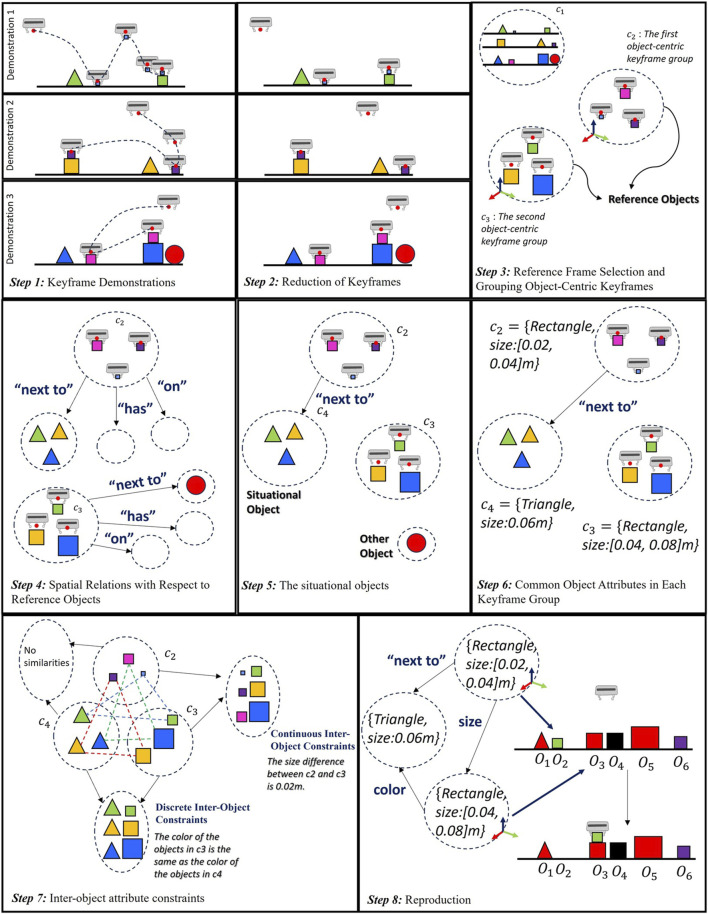
The steps of the proposed method from demonstration stage to reproduction stage.

This section introduces the proposed method, summarized graphically in Figure 2.

### 3.1 Demonstration of the task

The learning process commences once the user collects data from the robot through kinesthetic teaching, a method where the user directly manipulates the robot and upon guiding the robot to a desired pose, keyframes are recorded (Ravichandar et al., 2020). These keyframes are defined as descriptions of the end-effector pose, the poses of objects present in the scene, and the attributes of these objects, such as color, size, shape, etc. A demonstration is a sequence of keyframes, denoted as:

$$K_{i\;}=\left\{k_{1}^{i},k_{2}^{i},\ldots ,k_{j-1}^{i},k_{j}^{i},\ldots ,k_{m_{i-1}}^{i},k_{m_{i}}^{i}\right\},i\in \left[1,n\right]$$ (1)

Here, $K_{i\;}$ represents the set of keyframes for the *i*th demonstration. The variable *n* denotes the number of demonstrations, and *m*_*i*_ is the number of keyframes in the *i*th demonstration. This definition allows for a different number of keyframes for different demonstrations of the same task.

The term $k_{j}^{i}\in K_{i}$ represents the *j*th keyframe of the *i*th demonstration. $K_{i}$ is an ordered set, which means that $k_{j}^{i}$ is always recorded after $k_{j-1}^{i}$ . The *j*th keyframe of the *i*th demonstration is represented by:

$$k_{j}^{i}=\left\{{\left(s_{r}\right)}_{j}^{i},E_{j}^{i}\right\}$$ (2)

Here ${\left(s_{r}\right)}_{j}^{i}$ contains the end-effector pose and gripper status, and $E_{j}^{i}$ is an environment set that includes the set of object poses and object attributes in the *j*th keyframe of the *i*th demonstration. This representation allows for a comprehensive description of both the robot’s state and the state of the environment at each keyframe. The sets represented in Eq. 2 are further described as follows:

$${\left(s_{r}\right)}_{j}^{i}={\left\{x,y,z,q_{x},q_{y},q_{z},q_{w},\psi \right\}}_{j}^{i}$$ (3)

$$E_{j}^{i}={\left\{O_{1},O_{2},\ldots ,O_{\beta },{O_{\beta +1,}\ldots ,O}_{\tau_{i}}\right\}}_{j}^{i}$$ (4)

where $\left[x,y,z\right]\;\epsilon \;R^{3}$ represents the position of the end-effector, the orientation of end-effector is represented by unit quaternion’s real part $q_{w}\in R$ and vector part $\left[q_{x},q_{y},q_{z}\right]\in R^{3}$ , and ψ is a discrete value describing the gripper status. It can be either zero (open) or one (closed). In this set, $\tau_{i}$ represents the number of objects in each demonstration, a value that can change from one demonstration to another. For instance, as shown in Figure 2, while the first and second demonstrations include three objects, the last demonstration scene includes four objects. ${\left(O_{\beta }\right)}_{j}^{i}$ contains the attributes of the β -th object in the *j*th keyframe of the *i*th demonstration. This representation allows for a detailed description of each object’s characteristics within the demonstration.

### 3.2 Reduction of keyframes

The number of keyframes can change in every demonstration, as specified in Eq. 1. We categorize these recorded keyframes into two types: object-centric keyframes and task-unimportant keyframes. Object-centric keyframes are defined as those that alter an attribute in the environment set, while task-unimportant keyframes are considered as the remaining keyframes. Task-unimportant keyframes provide information on how to reach object-centric keyframes. As long as we prevent collisions, the method by which the robot reaches a goal state is not crucial for achieving the desired outcome.

In LfD, it is necessary to generalize the data obtained from multiple demonstrations to accommodate changes in task parameters, such as object poses and/or attributes. As discussed in the related work, traditional methods in keyframe demonstrations cluster similar robot motions using unsupervised learning or alignment methods (Akgun et al., 2012b). However, these methods do not elucidate the semantic similarity of these keyframes. For instance, the first step of Figure 2 presents several keyframe demonstrations for a stacking task. The user picks up an object and places it on top of another object. For example, in the first step of this figure, the fourth keyframe in the first demonstration is close to the releasing keyframe. This could lead to a cluster that includes both the releasing keyframes from each demonstration and this task-unimportant keyframe. However, it is necessary to have the same object-centric keyframes in a cluster. These keyframes provide the accurate relative poses between the end-effector and the object. By grouping these object-centric keyframes from different demonstrations, we can focus directly on the same relevant objects for each demonstration. Therefore, eliminating task-unimportant keyframes instead of using all the keyframes recorded by the user yields this outcome. We have the following assumptions:

• Each task necessitates a minimum number of keyframes, referred to as object-centric, to adequately summarize the task being learned by the robot.
• Users are expected to record at least the object-centric keyframes, and possibly more, namely, task-unimportant keyframes.

The recognition and elimination of task-unimportant keyframes from the data is a contribution of this work. Our aim is to identify object-centric keyframes from the set recorded by the user. The presence of any object in the environment set, as defined by Eq. 4, depends on relevant object poses and attributes, as follows:

$${\left(O_{\beta }\right)}_{j}^{i}={\left\{s_{O_{\beta }},\xi_{O_{\beta }}\right\}}_{j}^{i},\;\forall \beta \in \left[1,\tau_{i}\right]$$ (5)

$${\left(s_{O_{\beta }}\right)}_{j}^{i}={\left\{x,y,z,q_{x},q_{y},q_{z},q_{w}\right\}}_{j}^{i}$$ (6)

$${\left(\xi_{O_{\beta }}\right)}_{j}^{i}={\left\{\zeta_{O_{\beta }},A_{O_{\beta }}\right\}}_{j}^{i}$$ (7)

As indicated in Eq. 5, ${\left(s_{O_{\beta }}\right)}_{j}^{i}$ and ${\left(\xi_{O_{\beta }}\right)}_{j}^{i}$ represent the poses and attributes of object $O_{\beta }$ in the *j*th keyframe of the *i*th demonstration, respectively. $\zeta_{O_{\beta }}$ in the set of ${\left(\xi_{O_{\beta }}\right)}_{j}^{i}$ provides information about whether an object is held by the robot or is in the environment. We assume that if the robot manipulates the object, the value of $\zeta_{O_{\beta }}$ is “grasping”, oppositely its value is “on the table”. $A_{O_{\beta }}$ , which is defined in the next section, can be a combination of discrete (color, shape, etc.) and continuous (size, mass, etc.) attributes. It is important to note that $\zeta_{O_{\beta }}$ and $A_{O_{\beta }}$ can change in any keyframe of any demonstration. For instance, as seen in the first step of Figure 2, the object is held by the robot in the second keyframe of the first demonstration, while a similar object is held in the third keyframe of the second demonstration.

Object-centric keyframes necessitate changes in the object’s attribute set, denoted as ${\left(\xi_{O_{\beta }}\right)}_{j}^{i}$ , which is described in Eq. 7. Therefore, a reduced keyframe set is given by:

$$\left.{\hat{K}}_{i}=\left\{\left.k_{j}\right|\;k_{j}\in K_{i},{\left\{\xi_{O_{\beta }}\right\}}_{\left(j-1\right)}\neq {\left\{\xi_{O_{\beta }}\right\}}_{j},\forall O_{\beta }\in E_{j}^{i}\right\}\right.$$ (8)

The first keyframe, $k_{1}$ , is recorded before the user initiates the demonstration. After reducing keyframes, each demonstration has a similar number of keyframes, including object-centric ones. For instance, as depicted in the first step of Figure 2, the first keyframe is always retained. In the second keyframe of the second demonstration, the robot does not alter anything in the environment, so we can remove it. In the third keyframe of the second demonstration, the object is grasped, leading to an update in the reduced keyframe set ${\hat{K}}_{i}$ . After applying Eq. 8, as shown in the second step of Figure 2, we have three keyframes in each demonstration.

### 3.3 Reference frame selection

Each demonstration presents a unique scene, featuring a variety of objects and object poses. For successful reproduction, it’s crucial to generalize the robot’s motion to accommodate unseen objects and varying object poses. Relying solely on a single reference frame, such as the robot base frame, may not facilitate understanding the desired relative poses between the end-effector and objects. This is because object poses can vary from one demonstration to another. To effectively generalize the robot’s motion to a new environment and comprehend the relevant attributes of the objects, it’s important to use the pose of an object as a reference frame for each keyframe. In our scenario, after the reduction of keyframes, each object-centric keyframe from different demonstrations corresponds to an object. These keyframes are defined when there’s a change in an object’s attribute. Our algorithm takes into account three different situations:

• The robot may pick up an object.
• An object attribute can change in any keyframe, exemplified by the glass being full in one keyframe and empty in the previous one.
• The robot can place an object into target positions.

The selection of the reference frame for these three situations is detailed in Algorithm 1.

Algorithm 1 Selection of Reference frames and Reduction of Keyframes.

**Input**: $K_{i},m_{i},\tau_{i}$

**Output**: Reference frame, F_j_, for each reduced keyframes
***Initialize***: ${\hat{K}}_{i}\to \;{\left\{s_{r},E\right\}}_{i}^{1}$ F_j_ → {robot base frame}
***for*** j = 1: $m_{i}$ :*//For each keyframe in the ith demonstration*
 ***if*** Update ${\hat{K}}_{i}\to \;K_{i}$ using Eq. 8
*//If the attribute set has changed*
  ***for*** β *= 1:* $\tau_{i}$ *:*
   ***if*** ${\left(\zeta_{O_{\beta }}\right)}_{\left(j-1\right)}<\;{\left(\zeta_{O_{\beta }}\right)}_{j}$ or ${\left(A_{O_{\beta }}\right)}_{\left(j-1\right)}\neq {\left(A_{O_{\beta }}\right)}_{j},$

 *//The position of the object that is held or whose properties change*
    
$F_{j}\to \;O_{\beta }$

    **Break;**
   ***elseif*** ${\left(\zeta_{O_{\beta }}\right)}_{\left(j-1\right)}>\;{\left(\zeta_{O_{\beta }}\right)}_{j}$ or ${\left(s_{o_{\beta }}\right)}_{j-1}\neq {\left(s_{o_{\beta }}\right)}_{j}$ :
 *//The position of the object that is released or whose pose change*
    
$F_{j}\to \;computeSpatialRelations\left(O_{\beta },\tau_{i}\right)$

    **Break;**
   ***else***:
    
$F_{j}\to \;robot\;base\;frame$

   ***end***
  ***end***
 ***end***
***end***
***def*** $F_{j}=computeSpatialRelations\left(O_{\beta },\tau_{i}\right)$ :
 Initialize relation: {}
 count = 1
 flag = 0
 objects = $\left\{\left.1,2,\ldots ,\beta -1,\beta +1,\ldots ,\right.\tau_{i}\right\}$ *//Objects except for* $O_{\beta }$

 ***for*** k = 1: $\tau_{i}-1$ :
  ***if*** $O_{\beta }\;is\;on\;O_{\text{objects}\;\left(k\right)}:$

   
$relation\left(k\right)\to \;\left\{O_{objects\left(k\right),}{\;}^{\prime \prime }on^{\prime \prime }\right\}$

   flag = 1;
   break;*//If the released object is on another object, the function is ended*
  ***elseif*** $O_{\beta }\;is\;next\;to\;O_{objects\;\left(k\right)}:$

   
$relation\left(k\right)\to \;\left\{O_{objects\left(k\right)\;}{,}^{\prime \prime }nextto^{\prime \prime }\right\}$

 *//The released object is next to another object*
  ***else***:
   
$relation\left(k\right)\to \left\{O_{objects\left(k\right)}{,}^{\prime \prime }base^{\prime \prime }\right\}$

  ***end***
 ➢ If the flag = 1, $F_{j}\to O_{objects\left(k\right)\;}$

 ➢ Elseif find the index of next to label $F_{j}\to$ find object index in relation array that  has “next to” label
 ➢ Else $F_{j}\to robot\;base\;frame$

Although the reference frame is chosen as the object pose whose attributes change in a keyframe in the first two conditions, it’s crucial to understand the reasoning behind the robot’s action when it places an object in a target position. To achieve this, we compute the spatial relations between the picked objects and other objects in the environment. In this work, we primarily focus on the “on top/in” and “next to” situations. However, these semantic labels can be extended using relevant studies (Ziaeetabar et al., 2017) to other spatial relations for more precise results.

When a robot releases an object, we are not interested in the released object itself, but rather the reason behind this action. We analyse the position of the released object relative to others using two labels: “on top of” and “next to”. Let’s assume object A is placed in a target location with only one other object, i.e., object B. If A rests on top of B, object B becomes the reference object for that keyframe. Similarly, if A is dropped next to B, object B serves as the reference object. However, situations arise where the dropped object A might be both on top of object B and next to object C. In such cases, the algorithm prioritizes the “on top of” relationship and object B is still chosen as the reference object. Because the concept of “next to” is inherently subjective, depending on user preference or task. For instance, a 1 cm distance might not be considered “next to” when working with small objects, while a 10 cm distance might be in other tasks. Therefore, if the result indicates that an object is “next to” another, our algorithm proceeds with the computation of the reference frame. The algorithm checks all objects without any specific order; at first object C can be checked, but there can be a possibility for an “on top of” situation. As shown in the second step of Figure 2, the robot picks up an object in the second keyframe, and the pose of the grasped object is described as a reference frame. The robot then places this object on top of another object. Even though the pose of the released object has changed in the attribute set, the reference frame for the third keyframe must be relevant to the object in the targe pose. In the last demonstration, the larger rectangle is chosen as the reference object because it satisfies the more definitive “on top of” relation.

### 3.4 Constraints for object and end-effector relative poses

After reducing the keyframes, we ended up with a similar number of keyframes, *m*, in each demonstration. This allows us to group these keyframes from different demonstrations based on the keyframe index, *j*∈[1,*m*], as we ensure an equal number of keyframes. The purpose of collecting these keyframes is two-fold: to extract similar relative poses between the end-effector and objects, and to identify the attributes of each object within the group. We use the reduced keyframe set in Eq. 8 and group them as follows:

$$c_{p}=\left\{\begin{array}{l} \left\{E_{p}^{1},E_{p}^{2},\ldots ,E_{p}^{n}\;\right\},p=1\\ \underset{i=\left[1,n\right]}{⋃}\left\{{\left(s_{r}\right)}_{p}^{i},{\left(O_{\beta }\right)}_{p}^{i}\;\right\},\forall {\left(O_{\beta }\right)}_{p}^{i}\to \;F_{p},p\in \left[2,m\right] \end{array}\right.$$ (9)

Here, c_p_ is one of the groups that includes the keyframes representing the same relations between the robot and the objects. Each keyframe in the c_p_ must alter the environment’s attributes in the same way. For instance, if the object attribute changes from “on the table” to “grasping” in a keyframe, these keyframes are collected to the same group, c_p_. As depicted in the second step of Figure 2, the second keyframes show the “grasping” and the last keyframes show the “releasing”. Therefore, they can be combined in order as illustrated in the third step. In the initial keyframe group, we retain all object information in the scene. The reason for this is explained in Section 3.5. However, other keyframe groups only include the robot’s pose and the poses of objects associated with reference frames, along with their attributes.

In order to be able to generalize the task to new environments, the robot needs to understand the desired relative pose between the end-effector and relevant objects. The poses of the end-effector and the objects are recorded as specified in Eqs 3, 6, respectively. In object-centric keyframes, we expect the end-effector poses in each demonstration to be similar with respect to relevant objects. Therefore, averaging these relative poses results in the desired relative pose. Then in a new environment with different object poses, when we know the desired relative pose we can compute the desired end-effector poses easily. To compute the average of relative positions, we simply divide the summation of relative positions in each keyframe group by the demonstration number. However, averaging quaternions is non-trivial. In this work, quaternions will be averaged using the method proposed by Markley et al. (2007).

### 3.5 Object attribute constraints between/within demonstrations

Once we determine the desired relative distance between the end-effector and objects for each keyframe, the robot can successfully complete the task, even with variations in object poses. As long as the robot is aware of the relevant objects, learning the desired relative poses between the robot and these objects is sufficient for accurately executing the robot’s motion. However, our aim is to learn a broader range of tasks, including more abstract goals. These goals might pertain to the objects in the scene; for example, placing a small object on top of a larger one, or placing a cube next to a cylinder. Furthermore, users may want to demonstrate the similarity of objects within demonstrations; the goal could be to place cups next to plates of the same color. In this work, we aim for these goals to be learned by the robot without explicit instruction from the user. In this section, we describe how the robot can learn the similarities between and within demonstrations as a constraint to successfully achieve and semantically understand the task.

Before computing these constraints, we consider three different object types, similar to the approach proposed by Chella et al. (2006). However, our aim differs from theirs; we seek to understand the common attribute between used objects to generalize the user’s intention. The three different object categories are:

Reference Objects: These are objects that are relevant to each keyframe, and the poses of these objects are chosen as the reference frame for each keyframe. We assume only one reference object exists per keyframe within a demonstration.

Situational Objects: These are objects that have a spatial relation with respect to reference objects in the first keyframes. However, to define an object under this category, the same spatial relations must exist for each demonstration.

Other Objects: These are objects that are neither reference nor situational objects.

For example, consider a task as depicted in Figure 2, where the robot is required to pick up the rectangle that is next to the triangle and place it on top of the larger rectangle. In the third step, the smaller rectangle serves as the reference object for the second keyframe, and the larger rectangle is the reference object for the third keyframe. The smaller rectangle is next to the triangle in each demonstration, hence this triangle is defined as a situational object. In the last demonstration, the larger rectangle is also next to a circle. However, the spatial relation between the larger rectangle and this circle does not exist in each demonstration. As a result, this circle is categorized as an “other” object.

In Eq. 9, the first keyframe group includes all object attributes to find the situational objects. To extract situational objects we use the “on top,” “next to,” and “has” labels. For instance, if a situational object is on top of a reference object, we interpret this as “the reference object has a situational object”. Similarly, if a reference object is “next to or on top of” a situational object, we interpret these constraints as “a reference object is next to/on top of a situational object”. For these situational objects, another keyframe group is generated and added to the object-centric keyframes. However, this new keyframe group does not contain any information about the robot’s pose. While the robot motion is generated using object-centric keyframes, these new sets can define the task goals, but the robot’s goal poses are defined using reference objects. For instance, in the fifth step of Figure 2, the second and third keyframes provide information about the robot’s motion, but the fourth keyframe group does not. However, if the user’s intention is to pick up the rectangle next to the prism, it implies that these situational objects may also be involved in the task goal’s constraint.

In this study, object attributes can be both discrete and continuous attributes, and they are represented as follows:

$$A_{O_{\beta }}={\left\{{ϴ}_{d_{k}},{ϴ}_{c_{k}}\right\}}_{j}^{i}$$ (10)

In Eq. 10, ${ϴ}_{c_{k}}$ represents the value for a continuous attribute $c_{k}$ , such as size or mass and similarly ${ϴ}_{d_{k}}$ represents the discrete value for a discrete feature $d_{k}$ such as color, shape, or category. We assume that each attribute is independent of other attributes. Firstly, we determine from the data, whether constraints must be imposed on a particular object attribute or not. This allows the robot to better understand the task and which attribute of reference and situational object is relevant during the reproduction step. Considering that the number of groups of situational objects is q, and the number of groups of object-centric keyframes is m-1, the total number of keyframe groups is calculated as q + m-1. We compute the distance of each discrete attribute value between demonstrations; then the summation of absolute values of these distances is computed as follows:

$$\chi =\sum_{i=1}^{n}\left|{\left\{{ϴ}_{d_{k}}\right\}}_{j}^{i+1}-{\left\{{ϴ}_{d_{k}}\right\}}_{j\;}^{i}\right|,j\in \left[2,q+m-1\right]$$ (11)

If χ is zero, it implies that the attribute is identical across all demonstrations, and a constraint must be added for the specific keyframe group. If this is not the case, the value of this attribute does not impact the task and can be disregarded by the robot. As illustrated in the sixth step of Figure 2, the shape of the objects in each group is the same, so the shape is considered a constraint. However, the color of the objects varies in every demonstration, therefore it is not defined as a constraint.

However, there might not always be similarities between a particular attribute in each demonstration when considering only one object. For instance, the color of the object that the robot picks may vary from one demonstration to another. Nonetheless, there might be a constraint between object pairs within each demonstration, e.g., the object must always be placed on an object with the same color. To handle such constraints, we compute a difference vector for the discrete attributes of each object in one demonstration as follows:

$$\begin{array}{l} {\left\{{ϴ}_{d_{k}}\right\}}_{j=\left\{a,b\right\}}^{i}={\left\{{ϴ}_{d_{k}}\right\}}_{a}^{i}-\;{\left\{{ϴ}_{d_{k}}\right\}}_{b}^{i},\\ \forall \left\{a,b\right\}\in \left[2,m+q-1\right],a\neq b \end{array}$$ (12)

If there is a constraint between the objects used in one demonstration, the value of ${\left\{{ϴ}_{d_{k}}\right\}}_{j}^{i}$ must be equal for each demonstration, so the result of

$$\begin{array}{l} {\left\{{ϴ}_{d_{k}}\right\}}_{j=\left\{a,b\right\}}^{i+1}-\;{\left\{{ϴ}_{d_{k}}\right\}}_{j=\left\{a,b\right\}}^{i},\\ \forall i\in \left[1,n\right] \end{array}$$ (13)

needs to be zero. Otherwise, it implies that there are no object attribute constraints within the demonstration. If it is zero Eq. 12 will be another constraint in the reproduction to define each object. For instance, in the seventh step of Figure 2, the color of the triangle in the fourth keyframe group and the color of the rectangle in the third keyframe group varies in each demonstration. However, in each demonstration, the color of the triangle matches the color of the rectangle. This means that the color of the triangle and the color of the rectangle can be any color, but as a constraint, their colors must be the same. It is important to note that although the shape of reference objects is also the same, the shape of these objects does not vary in each demonstration. As this constraint is already computed between demonstrations using Eq. 11, we do not check the shape of these objects within the demonstration.

Similar reasoning applies to continuous object attributes. With continuous attributes, the robot may observe that the object to be picked always has a different size. However, determining whether these sizes are similar enough to impose a constraint requires a measure of similarity and a defined threshold. Therefore, we assume that the continuous attribute values are normally distributed, and we compute the standard deviation, σ , and mean, μ , values of the attributes for each object given in the same keyframe. For a continuous variable, the range of ${\left\{{ϴ}_{c_{k}}\right\}}_{\tau }$ , which falls within μ±2σ , is considered a constraint for the object, as long as $\sigma <w_{\max}$ . If the standard deviation of a continuous variable exceeds a threshold, $w_{\max}$ , then this feature is assumed to be unconstrained. Large variations in continuous attributes suggest that these attributes are not crucial for object selection. This threshold must be predefined. For example, in the seventh step of Figure 2, a size range is computed as a constraint for the size of each object, and $w_{\max}=2cm$ .

Similarly, we check the similarities of different object’s continuous attributes within the demonstration using the correlation coefficient. A correlation of −1 defines a perfect negative correlation and a value of 1 defines a perfect positive correlation. In this work, only inter-object attributes with an absolute correlation of 0.65 or more are considered to be constrained to one another. For example, in each demonstration, small objects can be placed on top of big objects; in this case, if there are two big objects and one small object in the environment satisfying the constraints, the robot cannot make a decision about which object will be used. In this work, the relation between continuous object attributes that are correlated is modeled using a linear relation, however, any other model can be used if preferred. Therefore, we represent a linear equation to define the constraint,

$${\left\{{ϴ}_{c_{k}}\right\}}_{a}^{i}=X\;{\left\{{ϴ}_{c_{k}}\right\}}_{b}^{i}+Y,\forall \left\{a,b\right\}\in \left[2,m+q-1\right],a\neq b$$ (14)

Where the vectors *X* and *Y* are learned through linear regression, and in reproduction, the relevant objects can be chosen using the constraint for task objects and other objects used within the demonstration. For example, in the seventh step of Figure 2, the size difference between the small rectangle and the larger rectangle is consistently 0.02 m in each demonstration, therefore the coefficients of *X* and *Y* are recorded as 0.02 and 1 respectively. These constraints are utilized during the reproduction stage.

In this method, it is up to the user whether similar object attributes are observed in all demonstrations or if the same inter-object constraints exist in each demonstration. Users must utilize the objects according to their purpose during demonstrations. It is not necessary for discrete or continuous attributes to always have constraints. For instance, if the user does not change objects but uses a different geometric configuration in each demo, our method only takes into account the observed objects and their attributes for the task.

### 3.6 Reproduction

The inputs of the reproduction include constraints for each keyframe group, between keyframes, the number of reference objects, and the new environment with object attributes and poses. The first step classifies objects based on desired attributes for each keyframe group. For example, in Figure 2, there are two reference objects and one situational object after demonstrations. Initially, six candidate objects exist for each keyframe in the reproduction scene. The first reference object must be a rectangle of size 0.02–0.04 m, with possible candidates being O_2_-O_6_. The second reference object must also be rectangular but of size 0.04–0.06 m, with candidates being O_3_-O_4_-O_5_-O_6_. The situational object is a triangle of size 0.06m; the only valid object is O_1_. A candidate object matrix is created, including all possible object index combinations for each keyframe. Columns of this matrix represent each keyframe group. Some examples of matrix rows are O_2_-O_3_-O_1_, O_2_-O_4_-O_1_, and O_6_-O_5_-O_1_. Constraints between keyframes are then checked. The order of this check doesn’t affect the final outcome, provided all constraints are considered.

First, constraints between the situational and reference object are applied. In the example of Figure 2, the first reference object must be next to the situational object. Relevant columns containing these objects are taken from the candidate object matrix, such as O_2_-O_1_ and O_6_-O_1_. Since O_2_ is next to O_1_, rows that include O_6_ in the first column are removed. Next, discrete object attribute constraints between keyframes are checked. The second reference object and situational object must have the same color. The rows that have the index of objects O_4_, O_6_ in the second column are removed, as they are not red. Lastly, continuous object attribute constraints are checked between keyframes. The second object’s size is twice the first reference object size. Since O_5_ is not valid for this constraint, the rows that include the index of O_5_ in the second column is removed. The result objects for each keyframes in order are O_2_-O_3_-O_1_. Ultimately, this process results in a single candidate object for each keyframe. The reference object number is crucial for computing the desired robot’s pose. In this example, the first two columns (O_2_-O_3_) are taken to compute the desired end-effector pose. This reproduction procedure is summarized in Supplementary Algorithm S1.

## 4 Experiments

### 4.1 Experimental setup

**FIGURE 3:**
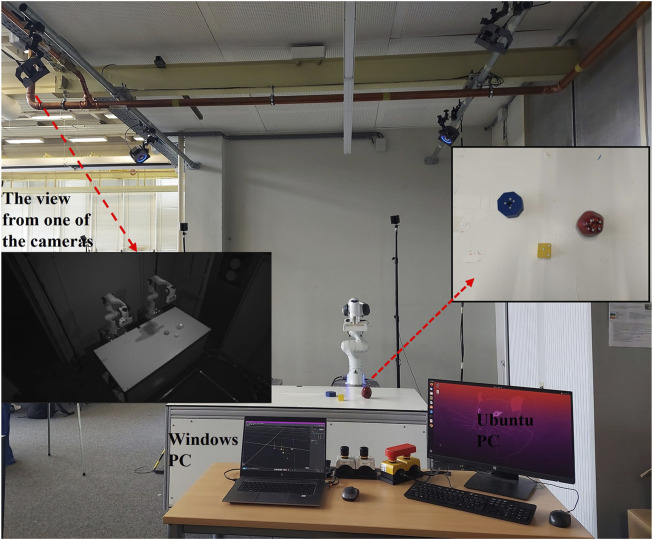
Experimental setup with a motion capture system. Multiple cameras are positioned around the table to capture the poses of the objects (geometric shapes) on the table. The inset shows a close-up view of the objects and markers. The view from one of the cameras is illustrated.

The Optitrack Motion Capture System, with four OptiTrack Prime 41/22 cameras, is used to detect object poses in the scene. The cameras are positioned to overlook the table during experiments. Their view, as illustrated in Figure 3, is captured using the Motive software on a Windows PC. The tracking of rigid bodies is done through the Motive software. A global frame is established during calibration, with a corner of the table designated as the global frame.

Passive reflective tape markers are used for object detection by the Optitrack system. Four markers were attached on top of each object due to their small size. The object pose is defined as the center of these markers. We consider five object attributes: color, shape, category, instance, and size. The “category” is a generic label for objects, such as toys or furniture. An “instance” of a category is a specific element within that category.

A 7-DOF Franka Emika Panda arm is used for manipulation tasks. To calibrate the robot’s base frame pose relative to the global frame, we use the OpenCV eye-to-hand library with the (Park and Martin, 1994).

During experiments, the user can move the robot freely. In each keyframe, the pose of the end-effector with respect to the robot base frame and the gripper positions are recorded through a Robot Operating System (ROS) node. The recorded data, including keyframe index, object poses, object attributes, and end-effector pose, are collected and used as input for the learning algorithm implemented in MATLAB. In the reproduction stage, the new environment information, including the pose of the objects in the scene and their attributes, and the generated constraints from the demonstration are input to Supplementary Algorithm S1. This algorithm identifies the relevant object for each keyframe in the scene and computes the result end-effector poses to satisfy the relative pose constraints between the robot and the object. We assume the existence of a trajectory planner that can generate a collision-free path between the desired end-effector poses.

### 4.2 Task descriptions

In our study, we validate our results using three tasks: stacking, sorting, and serving. Supplementary Videos demonstrating each experiment can be found in the Supplementary Materials as Video 1 (stacking), Video 2 (sorting), and Video 3 (serving), respectively. The task descriptions are given in the following lines.

• Stacking Task: This experiment demonstrates the robot’s ability to learn and generalize constraints about discrete object attributes. The goal is for the robot to *“Pick a cube and place it on top of a same-colored cylinder”.*
• Sorting Task: The second experiment emphasizes the importance of continuous object attribute constraints. The task is to *“Sort three objects of the same shape, each with a size difference of 0.01m from the next, from the largest to the smallest in right-to-left order.”*
• Serving Task: The final experiment highlights the relevance of situational objects and spatial constraints. The robot is taught to *“Choose another fruit from the box containing the same fruit as the fruit on the plate and place it next to the fruit on the plate.”*

## 5 Results

This section introduces the outcomes of the demonstrations and the results of reproduction for each individual tasks.

### 5.1 Stacking task

#### 5.1.1 Demonstration results

**FIGURE 4:**
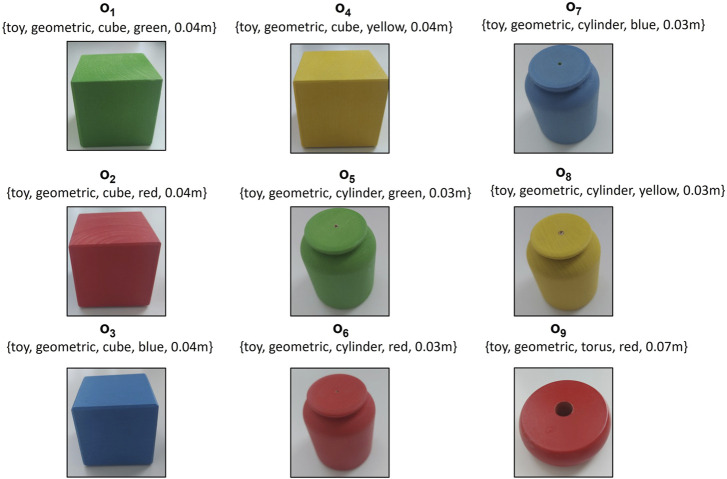
Variety of Geometric Objects for Stacking Task. Objects O1 (green cube), O2 (red cube), O3 (blue cube), and O5 (green cylinder), O6 (red cylinder), O7 (blue cylinder) are observed during the demonstration phase. Objects O4 (yellow cube), O8 (yellow cylinder), and O9 (red torus) are allocated for the reproduction phase.

**FIGURE 5:**
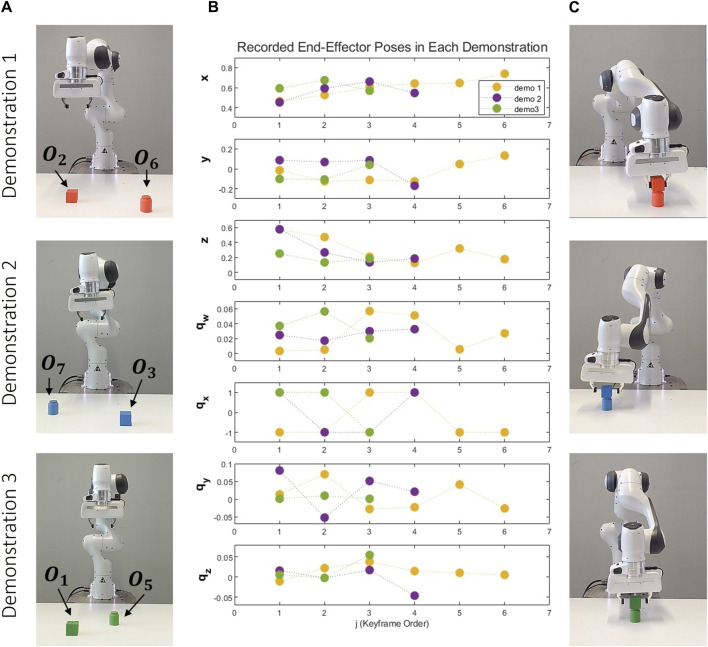
Demonstrations of the Stacking Task: This figure presents three distinct demonstrations, each arranged vertically and utilizing a unique set of objects. The first demonstration involves objects O2 and O6, the second incorporates objects O3 and O7, and the third employs objects O1 and O5. Each demonstration: **(A)** Depicts the initial scene before the demonstrations. **(B)** Graphically represents the recorded end-effector positions (x, y, z) and orientations (q_x_, q_y_, q_z_, q_w_) relative to the robot’s base frame. **(C)** Showcases the concluding object-centric keyframes.

During the learning phase of the stacking task, the robot consistently picks a cube and places it on top of a cylinder. The user varies the colour of the cube and cylinder in each demonstration. All objects utilized during the demonstration and reproduction phases are depicted in Figure 4. It’s important to note that not all objects are used during the training phase. Some are introduced in the reproduction phase to validate the generalization capabilities of our proposed approach. As depicted in Figure 5, three distinct demonstrations were executed. The initial demonstration involved a red cube and a red cylinder, the subsequent demonstration incorporated a blue cube and a blue cylinder, and the final demonstration utilized a green cube and a green cylinder. The configurations of these objects within each demonstration are illustrated in Figure 5A. Figure 5B provides a graphical representation of the end-effector positions and orientations, with the number of keyframes in each demonstration being 6, 4, and 3, respectively. Lastly, Figure 5C presents the scenes corresponding to the final object-centric keyframes in each demonstration.

We define the variable ζ based on the gripper status. At the start of each demonstration for a scene, its value is set to “on the table”. If the gripper status changes from open to closed in a keyframe, ζ is defined as “grasping.” Conversely, if it changes from closed to open, ζ is defined as “on the table”. In this example, only the positions of the cube and ζ change. Therefore, the reference frame for the second keyframe is the cube object location. Then, ζ changes from “grasping” to “on the table” for the cube. In this situation, our proposed method identifies a reference frame using other objects in the scene. Since the cube is on top of the cylinder, the reference frame for the third keyframe is defined as the cylinder location, as outlined in Algorithm 1. The first keyframe group,c_1_, represents the recorded scene before the demonstration, it is not important in learning to generalize the robot’s motion, its reference is taken.

As the robot base frame. After we apply Eq. 8, in this task, we only have two object-centric keyframes. The attributes for each keyframes are computed as follows:

$$c_{2}:\left\{\text{toy},\text{geometric},\text{cube},0.04m,\text{grasping}\right\}$$

$$c_{3}:\left\{\text{toy},\text{geometric},\text{cylinder},0.03m,\text{on the table}\right\}$$

Given that there is no constraint on the colour of the two objects, Eq. 12 has been used to establish a colour constraint between the objects, denoted as **c**_**2**_ **–c**_**3**_: {Colour}. More specifically, the colours of the object to be picked and the object on which this object must be placed are always identical. Since the environment only includes reference objects, there are no spatial constraints.

The size of the objects remains consistent in each demonstration. For continuous attributes, we always examine the correlation between two objects. However, there isn’t enough variation in size to determine whether a correlation exists. Therefore, size is not considered an inter-object constraint between two objects.

#### 5.1.2 Reproduction results

**FIGURE 6:**
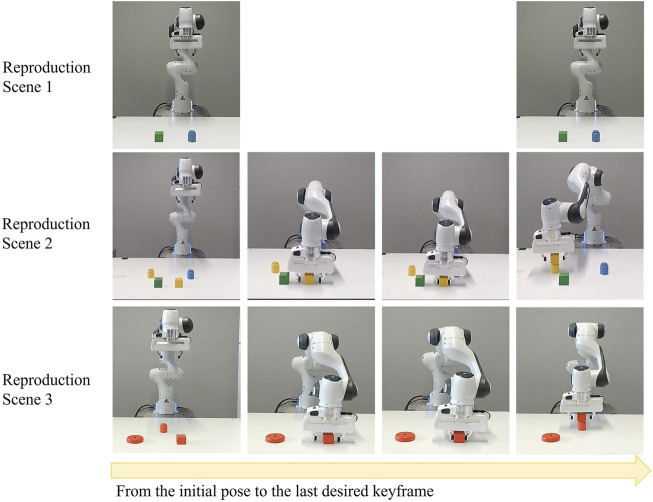
Reproduction Scenarios for the Stacking Task: This figure presents three distinct scenarios. Scene 1: an invalid configuration with a green cylinder and blue cube. Scene 2: a yellow cylinder and cube, and a blue cylinder and green cube, with the robot successfully stacking the yellow objects. Scene 3: a red cylinder, cube, and torus, with the robot stacking the red cube on the cylinder. Each column sequence illustrates the robot’s motion.

Following the teaching phase, the reproduction phase for this task takes the desired object attributes for each keyframe, discrete inter-object constraint, and the desired relative poses between the end-effector and reference objects as inputs. In the reproduction phase, we apply the learned constraints in three different environments, as depicted in Figure 6. In the first case, a green cylinder and a blue cube are present on the table. Although these objects meet the desired object attributes for each keyframe, the colour of the objects must be identical. Therefore, there are no suitable candidate objects for the task. In the second case, there are four objects. While the yellow cube and green cube are candidate objects for the second keyframe, the yellow cylinder and blue cylinder are chosen for the third keyframe. Since the colour of both objects must be the same, after the inter-object constraints in the reproduction steps, the resulting candidate objects are the yellow cube and yellow cylinder.

In the third case, a red cube, a red cylinder, and a red torus are present. Since the torus shape is not suitable for any keyframes, it is eliminated after applying the desired constraints for each keyframe.

The second and third cases are successful because there are objects that satisfy the constraints between demonstrations and discrete inter-object constraints. The outcomes of these two scenes are illustrated in the last column of Figure 6. The object attributes and the candidate objects in each environment are provided in Supplementary Table S1.

This result proves that the robot does not need task-unimportant keyframes to execute the task. Moreover, using only three demonstrations, we could extract the user’s intention. In reproduction, although there are multiple and unseen objects, the robot could generalize the task. The learning of discrete object attributes within the demonstrations indeed underscores the potential of our contribution. Without this contribution, the robot would execute the first case in the reproduction phase, even though it does not meet the user’s expectations.

### 5.2 Sorting task

#### 5.2.1 Demonstration results

**FIGURE 7:**
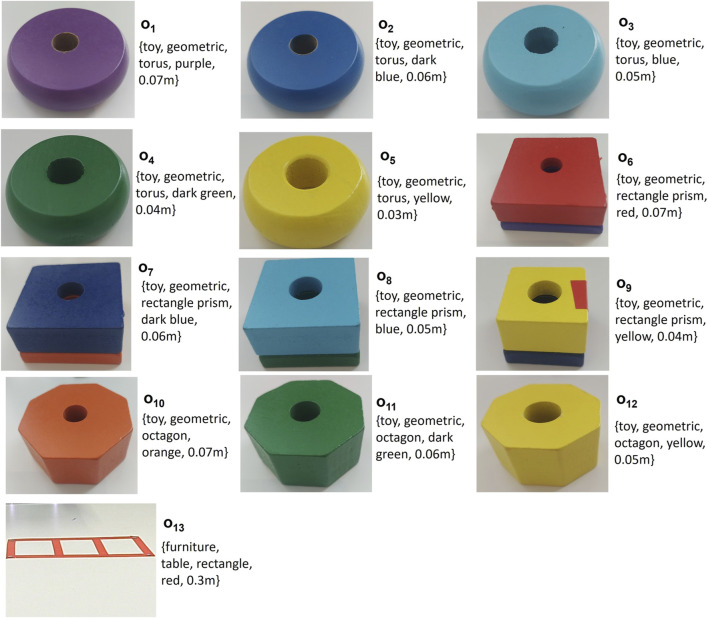
Variety of Geometric Objects for Sorting Task. Objects O_1_ (purple torus), O_2_ (dark blue torus), O_3_ (blue torus), O_4_ (dark green torus), O_5_ (yellow torus), O_7_ (dark blue rectangle prism), O_8_ (blue rectangle prism), O_9_ (yellow rectangle prism), O_13_ (red table) are observed during the demonstration phase. Objects O_6_ (red rectangle prism), O_10_ (orange octagon), O_11_ (dark green octagon), O_12_ (yellow octagon) are added for the reproduction phase.

Three demonstrations of the sorting task are sufficient to achieve this task. The objects used in the training and reproduction are illustrated in Figure 7.

**FIGURE 8:**
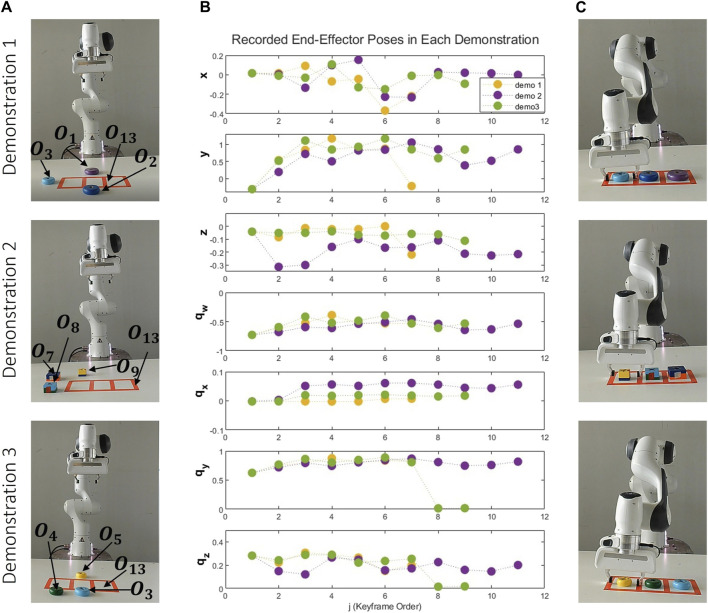
Demonstrations of the Sorting Task: This figure presents three distinct demonstrations, each arranged vertically and utilizing a unique set of objects. The first demonstration involves objects O_1_, O_2_, O_3_, and O_13_, the second incorporates objects O_7_, O_8_, O_9_ and O_13_, and the third employs objects O_3_, O_4_, O_5_, and O_13_. Each demonstration: **(A)** Depicts the initial scene before the demonstrations. **(B)** Graphically represents the recorded end-effector positions (x, y, z) and orientations (q_x_, q_y_, q_z_, q_w_) relative to the robot’s base frame. **(C)** Showcases the concluding object-centric keyframes.

As depicted in Figure 8, three distinct demonstrations were executed. Each environment used in the demonstrations contains three objects with the same shape and a red rectangular area for arranging these objects. The shapes and sizes of the objects to be sorted vary between demonstrations. The configurations of these objects within each demonstration are illustrated in Figure 8A. Since shape is a discrete attribute, using two different shapes is enough to teach that shape is not a significant factor between demonstrations. Figure 8B provides a graphical representation of the end-effector positions and orientations, with the number of keyframes in each demonstration being 7, 11, and 9, respectively.

Lastly, Figure 8C presents the scenes corresponding to the final object-centric keyframes in each demonstration. After calculating the constraints both between and within demonstrations, it’s found that the shape of objects within demonstrations is consistent. The size difference between two objects to be picked in order is 0.01 m in the same scene. The size of the largest object ranges between 0.08 m and 0.04 m, the second-largest object’s size ranges between 0.07 m and 0.03 m, and the smallest object’s size ranges between 0.06 m and 0.02 m. There are six object-centric keyframes, and the constraints for each keyframe are as follows:

$$c_{2}:\;\left\{\text{toy},\;\text{geometric},\;\left[0.04m,\;0.08m\right],\;\text{grasping}\right\}$$

$$c_{3}:\;\left\{\text{furniture},\;\text{table},\text{red},\;\text{rectangle},\;0.3m,\;\text{on the table}\right\}$$

$$c_{4}:\;\left\{\text{toy},\;\text{geometric},\;\left[0.03m,\;0.07m\right],\;\text{grasping}\right\}$$

$$c_{5\;}:\left\{\text{furniture},\;\text{table},\;\text{red},\;\text{rectangle},\;0.3m,\;\text{on the table}\right\}$$

$$c_{6}:\left\{\text{toy},\;\text{geometric},\;\left[0.02m,\;0.06m\right],\;\text{grasping}\right\}$$

$$c_{7}:\;\left\{\text{furniture},\;\text{table},\;\text{red},\;\text{rectangle},\;0.3m,\;\text{on the table}\right\}$$

Our analysis indeed goes beyond examining similar attributes of just two objects. For instance, this task involves four objects. When a discrete constraint exists between multiple keyframes within a demonstration, we combine these constraints in order. This approach allows for the faster elimination of possible incorrect objects during reproduction. A discrete constraint occurs when there is an equality. The discrete constraints between each keyframe group are defined as follows:

$$c_{2}\;–\;c_{4}\;–\;c_{6}:\;\left\{\text{Shape}\right\}$$

The shape of the reference objects in the second, fourth, and sixth keyframes must be the same. When continuous constraints of more than two keyframes exist, we separate them. This separation is necessary because we use a linear regression model, where one variable is the independent variable, and we always take the previous keyframe as the independent variable. The next keyframe, therefore, must be the dependent variable. Consequently, for continuous constraints, we combine each keyframe group as a pair, instead of a combination of them like discrete constraints. For example, in this task, a correlation exists between the c_2_-c_4_, c_4_-c_6_, and c_2_-c_6_ keyframe groups. Instead of recording all three constraints, we only record the c_2_-c_4_ and c_4_-c_6_ constraints. For example, in this task, shape is a discrete inter-object constraint between three objects, so we record these constraints as c_2_-c_4_-c_6_, which also reduced the data size used in reproduction. As a result, continuous attribute constraints between keyframes are,

$$c_{2}\;–\;c_{4}:\;\left\{\text{Size }\to \;\left[-0.01,1\right]\right\}$$

$$c_{4}\;–\;c_{6}:\;\left\{\text{Size }\to \;\left[-0.01,1\right]\right\}.$$

#### 5.2.2 Reproduction results

**FIGURE 9:**
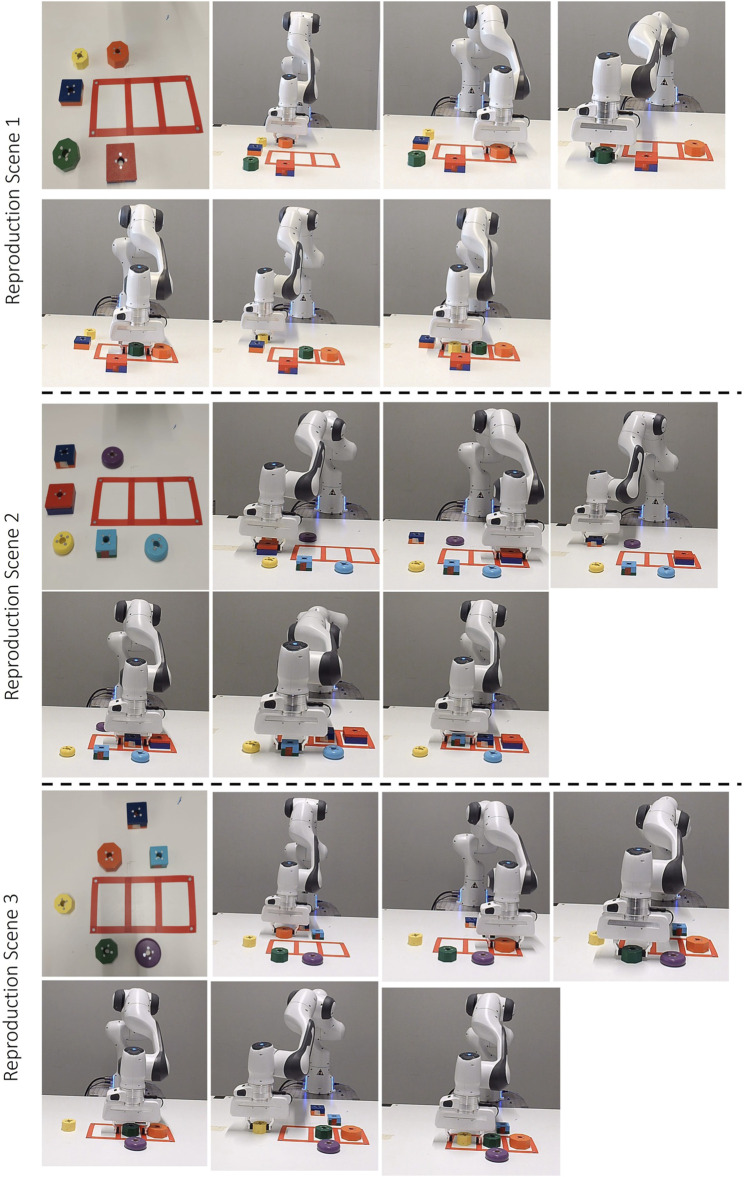
Reproduction Scenarios for the Sorting Task: This figure presents three distinct scenarios. Each scene is valid for the task’s description. Each column sequence illustrates the robot’s motion in each scene.

In reproduction, the robot executes three different scenarios as shown in Figure 9. The scenes are entirely new to the robot. While some objects have been seen in demonstrations, others, such as the octagon shapes, are new. During the demonstration and in the reproduction, we record objects using indexes. The robot does not receive any hints about the order of objects to be selected as a reference frame. Since only reference objects were used during demonstrations, the learning outcome does not include any constraints related to spatial relations. The input for reproduction consists of desired object attributes for each keyframe, discrete inter-object constraints, continuous inter-object constraints, desired relative poses between the end-effector and objects, and the new environment’s object attributes and poses. The candidate objects of each keyframe for each case are summarized in Supplementary Table S2.

In the first environment, after applying constraints between demonstrations, the candidate objects for the c_2_-c_4_-c_6_ groups can be any toy in the environment. However, the objects for c_3_-c_5_- c_7_ are red rectangle area on the table. These possible object sets will be decreased by applying discrete constraints within the task. This step eliminates the rectangle prism objects since the robot requires three objects with the same shape. However, for c_2_-c_4_-c_6_, there are still multiple possible objects. Next, continuous inter-object constraints are applied. When the robot chooses three objects in the correct order, it satisfies the task objectives. Therefore, the task is executed successfully. In fact, the dark green octagon size falls within the required size range for the largest object. However, if the robot takes this object as the largest, the robot cannot find any object for the smallest one. Consequently, it learns to eliminate these options.

In the second scenario, although the shape of all objects satisfies inter-object discrete constraints, the size difference between the second and the third object is 0.02 m instead of 0.01 m. Therefore, the robot chooses rectangle prisms. In the last scenario, the sizes of objects are suitable for the task. However, there are no three rectangle prisms, therefore the robot chooses octagons.

This result indeed demonstrates that continuous inter-object constraints can also be crucial for the task. Without this contribution, the robot would not be able to learn the size difference between the objects. Moreover, focusing on only object-centric keyframes assists in dealing with multiple objects, as each object is relevant to a keyframe.

### 5.3 Serving task

#### 5.3.1 Demonstration results

**FIGURE 10:**
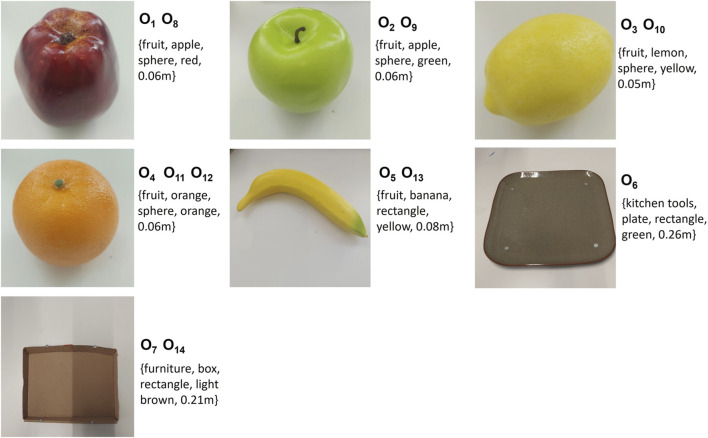
Variety of Objects for Serving Task. Objects O_1_ (red apple), O_8_ (red apple), O_2_ (green apple), O_9_ (green apple), O_3_ (lemon), O_10_ (lemon), O_5_ (banana), O_7_ (brown box), O_6_ (green plate) are observed during the demonstration phase. Objects O_4_, O_11_, O_12_ (orange), O_13_ (banana), O_14_ (brown box) are added for the reproduction phase.

In this task the fruit on the plate and the fruit in the box are the same. So, multiple objects with the same attributes can be on the table at the same time, these objects are represented with different indexes, as depicted in Figure 10.

**FIGURE 11:**
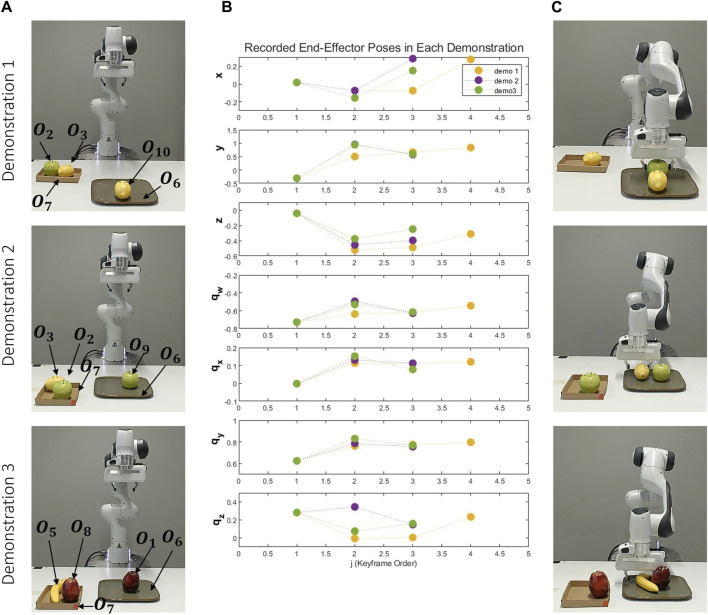
Demonstrations of the Serving Task: This figure presents three distinct demonstrations, each arranged vertically and utilizing a unique set of objects. The first demonstration involves objects two lemons, a green apple, a box and a plate. The second incorporates objects two green apples, one lemon, a plate and a box, and the third employs objects two red apples, one banana, a box and a plate. Each demonstration: **(A)** Depicts the initial scene before the demonstrations. **(B)** Graphically represents the recorded end-effector positions (x, y, z) and orientations (q_x_, q_y_, q_z_, q_w_) relative to the robot’s base frame. **(C)** Showcases the concluding object-centric keyframes.

As depicted in Figure 11, three distinct demonstrations were executed. As illustrated in Figure 11A, in the first demonstration, there’s a lemon on the plate and a lemon and a green apple on the box. The second demonstration has a green apple on the plate, and a lemon, and another green apple on the box. The third demonstration has a red apple on the plate, and a red apple and a banana on the box. Figure 11B graphically represents the end-effector’s positions and orientations, with each demonstration consisting of 4, 3, and 3 keyframes, respectively. Finally, Figure 11C visually presents the scenes associated with the last object-centric keyframes for each demonstration. There are two different reference objects and two object-centric keyframes for these objects. The constraints between these keyframes groups are:

$$c_{2}:\left\{\text{Fruit},\left[0.03,0.09\right]m,\text{grasping}\right\}$$

$$c_{3}:\left\{\text{Kitchen Tool},\text{Plate},\text{Green},\text{Rectangle},0.26m,\text{on the table}\right\}$$

In this task, there are situational objects that are automatically defined from the data. The labels for these spatial constraints are as follows: **c**_**2**_ is on top of **c**_**4**_, **c**_**2**_ is next to **c**_**5**_, and **c**_**3**_ has **c**_**6**_. As a result, the number of keyframes’ group has increased. The common attributes in each new keyframe group are:

$$c_{4}:\left\{\text{Furniture},\text{Box},\text{Light Brown},\text{Rectangle},0.21m,\text{on the table }\right\}$$

$$c_{5\;}:\left\{\text{Fruit},\text{Sphere},\left[0.04,0.07\right]m,\text{on the table }\right\}$$

$$c_{6\;}:\left\{\text{Fruit},\text{Sphere},\left[0.04,0.07\right]m,\text{on the table }\right\}$$

The first reference object (the fruit to be picked) is on the box and next to another fruit. Moreover, the second reference object (plate) has a fruit. The instance of the fruit next to the first reference object is the same as the instance of the fruit on the second reference object. This is computed as the discrete constraints between the fifth and sixth keyframe groups:

$$c_{5}‐\;c_{6}:\left\{\text{Colour},\text{Instance Of}\right\}$$

Moreover, since the size of the two objects in the fifth keyframe and sixth keyframe is the same, a continuous attribute constraint is found between them:

$$c_{5}‐\;c_{6}:\left\{\text{Size }\to \;\left[0,1.0\right]\right\}$$

Indeed, while the proposed method initially seeks situational objects using spatial constraints for each task, in previous experiments, all objects in the scene were reference objects.

However, the user’s intention may also depend on situational objects in the environment. Consequently, all these objects are added as additional groups. It’s important to note that the end-effector goal poses depend solely on reference objects. Situational objects merely assist in selecting these reference objects.

#### 5.3.2 Reproduction results

**FIGURE 12:**
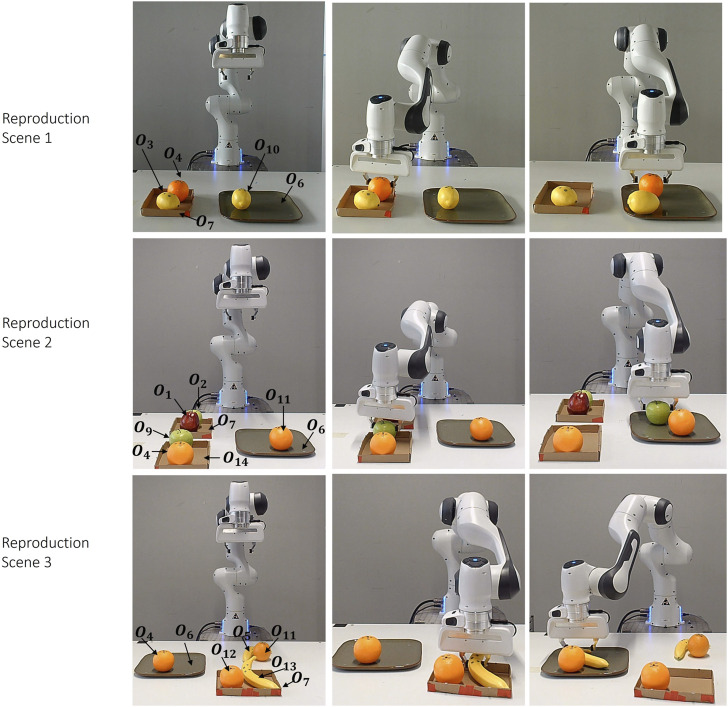
Reproduction Scenarios for the Serving Task: figure presents three distinct scenarios. Scene 1: a lemon is on the plate, an orange and a lemon are on the box. Scene 2: There are two boxes. A red apple and a green apple are on one of the box. A green apple and an orange are on the other box. An orange is on the plate. Scene 3: A banana and an orange are on the table. A banana and an orange on the box, and an orange on the plate. Each column sequence illustrates the robot’s motion.

In the reproduction phase, the robot performs the task in three different environments, as depicted in Figure 12. In the first scenario, the robot encounters a new fruit, an orange, which is on the box. A lemon is situated next to the orange, and another lemon is on the plate. Since the two situational objects must be the same, the lemons are situational objects. The robot selects the orange as a reference object, with the plate being the other reference object.

In the second scenario, the complexity is increased with the presence of two boxes, each containing two fruits, and an orange on the plate. The orange on the plate serves as one of the situational objects. The robot then looks for the same situational object (another orange) on the box. As a result, the robot selects the box containing an orange and green apple, successfully picking up the green apple.

In the final scenario, there are two fruits (a banana and an orange) on the box, two fruits (a banana and an orange) on the table, and an orange on the plate. The orange on the plate is one of the situational objects, but there is one orange on the table and one orange on the box. Even though both fruits are next to a banana, the banana must be on the box due to the spatial constraints between c_2_ and c_4_. Therefore, the robot picks up the banana on the box. The resulting candidate objects for each keyframe are provided in Supplementary Table S3.

However, due to the spherical shape of the fruits in c_5_ and c_6_, if there is a banana on the plate, the reproduction will fail. Of course, this situation can be solved by an additional demonstration with the banana placed on the plate. However, as we mentioned before, obtaining these constraints requires the teacher to make demonstrations that meet their own intentions.

Indeed, this experiment demonstrates the power of keyframe representations in handling situations where the user’s intentions may be associated with situational objects. Without this contribution, the robot might choose any fruit without considering its location or the fruit on the plate, thereby not fully meeting the user’s expectations. This approach ensures that the robot’s actions align more closely with the user’s intentions, showcasing the effectiveness of our method.

## 6 Discussion and conclusion

This study employs keyframe demonstrations to capture user intentions addressing two distinct challenges. The first challenge involves extracting object-centric keyframes from a varying number of keyframes in each demonstration. The second challenge pertains to inferring the user’s intention based on the attributes of objects and generalizing this understanding to environments that have not been previously encountered.

In the existing literature, the number of keyframes is often treated as equal (Jankowski et al., 2022) or even when the number of keyframes varies, the task is simple enough to involve only one object (Akgun et al., 2012b; Akgun and Thomaz, 2016). These keyframes, obtained from multiple demonstrations, must be clustered to generalize robot motion to different configurations. This clustering is dependent on the performance of alignment and clustering methods. In our study, we diverge from previous research by expanding the keyframe to include attributes of all objects in the environment, their poses, and the robot’s configuration. This additional information identifies which keyframes are necessary for the task, ensuring an equal number of keyframes are obtained in each demonstration. As it is easier to group equal numbers of keyframes, each with the same meaning, it eliminates the need for the alignment method, unlike other studies. This elimination guarantees keyframe groups that include accurate relative poses between the end-effector and objects, without being affected by task-unimportant keyframes which may cause coarser relative poses than the user intended. Our experiments indicate that when the number of keyframes changes in each demonstration, only object-centric keyframes are sufficient to reproduce the task. Moreover, this reduction of keyframes aids in addressing the second challenge.

The second challenge is to uncover the user intention about the attributes of objects to generalize the robot’s task to unseen environments. In the literature, studies such as those conducted by Cubek et al. (2015), focus solely on pick-and-place tasks involving two objects. They examine the similarities of object attributes between demonstrations. Similarly, in the study conducted by Chao et al. (2010), the similarities of both discrete and continuous attributes between demonstrations are given. These studies do not address similarities within a demonstration, such as the color of two objects having to be the same in a demo. Our solution can handle these situations. For example, in the first and second examples, the user desires depend on both similarities between and within the demonstration.

In the studies carried out by Fonooni et al. (2016), a semantic network includes all the necessary concepts and objects that the robot can work with, and nodes of the semantic network are discrete. However, in our experiments, to show that the shape of objects is not important, we do not have to include all possible shapes that the robot can work within our attributes. Moreover, the approach proposed by Fonooni et al. (2016), does not include similarity for continuous attributes. In the second experiment, we show the importance of continuous object attributes both between and within demonstrations. The size difference between each object can be important for a sorting task. These previous studies (Chao et al., 2010; Cubek et al., 2015; Fonooni et al., 2016) do not focus on the attributes of situational objects in the environment. For example, in the last experiment, we showed that for a serving task, the fruit on the box and on the plate must be the same. This constraint is determined using situational objects’ attributes. In summary, the similarities between object attributes are revealed in a more comprehensive way with complicated tasks involving multiple objects than the studies in the literature by taking advantage of keyframes. The constraints obtained as a result of this solution determine the purpose of the task, and the robot successfully performs the task by finding objects that meet these constraints, even if they are objects it has not seen before.

As discussed in the related work, the concept of semantics can be associated with a variety of applications. Our approach, when applied to object search applications such as those conducted by Guo et al. (2022) and Zhang et al. (2023), has certain limitations, particularly in the representation of object attributes. We demonstrate that if discrete object attributes match, then this attribute becomes a constraint. However, in the case of a service robot, the object could be located anywhere, necessitating a discrete object attribute to define its location. For instance, if a user demonstrates to the robot how to pick up a mug from the kitchen, the robot may not be able to find the mug if it is in another room during reproduction. Typically, navigation or object search applications use ontology-based graph representation, which could enhance our approach.

When applied to semantic navigation applications like Qi et al. (2020) and Achat et al. (2023), another limitation arises. We use keyframes to represent robot targets, but they may not generate constraints like “avoid the walls”. Adding trajectories could provide these constraints. Some studies propose hybrid movements as input (Akgun et al., 2012a), where both keyframes and trajectories can be provided. We aim to enhance our approach to accept trajectories as input in future work.

We believe that our approach has potential for applications in human-robot interaction. Although natural language is user-friendly, as shown in studies by Kartmann et al. (2021) and Bucker et al. (2023), these applications typically require a mapping from sentences to low-level robot motion and generally require substantial data. With our approach, users can implicitly teach their high-level preferences to the robot through physical interactions. Another potential application could be semantic grasping, as demonstrated by Moon and Lee (2020), if we add attributes such as “fragile” and “not fragile”, and record forces by the robot. Physical human-robot interactions when the robot executes the task can be another application that could benefit from our approach. For example, when there is a misalignment between the user and robot, the user records multiple trajectory traces around the object, as shown in the study by Lourenço et al. (2023). With the use of our approach, the user could show the trajectories around “computer,” “mobile phone” and the robot could understand implicitly that it needs to avoid “electronic devices”.

While not explored in this study, our method can effectively communicate the reasons for task failures to the user when the robot cannot find a solution for a task. For instance, in the first experiment, when there was a green cylinder and a blue cube, the robot would not perform the task because the colors did not match, thus not meeting the user’s intentions. In fact, the robot can understand the reason for failure, and these reasons can be informative for the user. The user can rectify these situations by providing an additional demonstration or changing objects based on the feedback received.

Similarly, in some contexts, there may be more than one group of candidate objects that meet the constraints derived from demonstrations. In fact, our proposed method can accurately identify all possible candidate objects. If combined with behavior trees (Gustavsson et al., 2022), the robot motions can be repeated until there are no objects in the environment that satisfy the desired constraints. However, we do not allow the robot to repeat the task when there are multiple correct object groups, as this may be incorrect for some tasks. For example, in the scenario discussed in the first experiment, when there is a red cylinder, red cube, yellow cylinder, and yellow cube, our algorithm provides us with the object pairs and the necessary keyframes and reference poses of the robot. However, when we apply this situation to a sorting task, the red area will already be full after the robot executes the task once. By avoiding the use of an object more than once for the same conceptual keyframe, we can easily handle this problem. But this will not be able to solve the situation of collecting fruits in a box, where we can put multiple fruits into a box. Although the message we want to convey can be understood by the robot, there may be situations that the robot cannot cope with. Asking the user about these situations through communication and after getting approval from him/her may perhaps take our work one step further. We believe that human-robot interaction is crucial to correcting the robot’s behavior or updating the task.

## Data Availability

The original contributions presented in the study are included in the article/Supplementary Material, further inquiries can be directed to the corresponding author.

## References

[B1] Ab Azar N. Shahmansoorian A. Davoudi M. (2020). From inverse optimal control to inverse reinforcement learning: a historical review. Annu. Rev. Control 50, 119–138. 10.1016/j.arcontrol.2020.06.001

[B2] Achat S. Serdel Q. Marzat J. Moras J. (2023). A case study of semantic mapping and planning for autonomous robot navigation. SN Comput. Sci. 5, 55. 10.1007/s42979-023-02370-3

[B3] Akgun B. Cakmak M. Jiang K. Thomaz A. L. (2012a). Keyframe-based learning from demonstration: method and evaluation. Int. J. Soc. Robot. 4, 343–355. 10.1007/s12369-012-0160-0

[B4] Akgun B. Cakmak M. Yoo J. W. Thomaz A. L. (2012b). “Trajectories and keyframes for kinesthetic teaching: a human-robot interaction perspective,” in *Proceedings of the seventh annual ACM/IEEE international conference on Human-Robot Interaction*, (ACM), 391–398. 10.1145/2157689.2157815

[B5] Akgun B. Thomaz A. (2016). Simultaneously learning actions and goals from demonstration. Auton. Robots 40, 211–227. 10.1007/s10514-015-9448-x

[B6] Arora S. Doshi P. (2021). A survey of inverse reinforcement learning: challenges, methods and progress. Artif. Intell. 297, 103500. 10.1016/j.artint.2021.103500

[B7] Avaei A. Van Der Spaa L. Peternel L. Kober J. (2023). An incremental inverse reinforcement learning approach for motion planning with separated path and velocity preferences. Robotics 12, 61. 10.3390/robotics12020061

[B8] Billard A. Calinon S. Dillmann R. Schaal S. (2008). “Robot programming by demonstration,” in Springer handbook of robotics. Editors Siciliano B. Khatib O. (Berlin, Heidelberg: Springer Berlin Heidelberg), 1371–1394. 10.1007/978-3-540-30301-5_60

[B9] Bobu A. Bajcsy A. Fisac J. F. Dragan A. D. (2018). “Learning under misspecified objective spaces,” in Conference on robot learning (Zurich, Switzerland: PMLR), 796–805.

[B10] Bucker A. Figueredo L. Haddadin S. Kapoor A. Ma S. Vemprala S. (2023). “LATTE: LAnguage trajectory TransformEr,” in 2023 IEEE international conference on robotics and automation (ICRA) (London, United Kingdom: IEEE), 7287–7294. 10.1109/ICRA48891.2023.10161068

[B11] Bucker A. Figueredo L. Haddadinl S. Kapoor A. Ma S. Bonatti R. (2022). “Reshaping robot trajectories using Natural Language commands: a study of multi-modal data alignment using transformers,” in 2022 IEEE/RSJ international conference on intelligent robots and systems (IROS) (Kyoto, Japan: IEEE), 978–984. 10.1109/IROS47612.2022.9981810

[B12] Bullard K. Akgun B. Chernova S. Thomaz A. L. (2016). “Grounding action parameters from demonstration,” in 2016 25th IEEE international symposium on robot and human interactive communication (RO-MAN) (New York, NY, USA: IEEE), 253–260. 10.1109/ROMAN.2016.7745139

[B13] Calinon S. (2018). “Learning from demonstration (programming by demonstration),” in Encyclopedia of robotics. Editors Ang M. H. Khatib O. Siciliano B. (Berlin, Heidelberg: Springer Berlin Heidelberg), 1–8. 10.1007/978-3-642-41610-1_27-1

[B14] Chao C. Cakmak M. Thomaz A. L. (2010). “Interactive task learning with discrete and continuous features,” in Visual representations and reasoning.

[B15] Chella A. Dindo H. Infantino I. (2006). “Learning high-level tasks through imitation,” in 2006 IEEE/RSJ international conference on intelligent robots and systems (Beijing, China: IEEE), 3648–3654. 10.1109/IROS.2006.281721

[B16] Chernova S. Thomaz A. L. (2014). Robot learning from human teachers. Cham: Springer International Publishing. 10.1007/978-3-031-01570-0

[B17] Cubek R. Ertel W. Palm G. (2015). “High-level learning from demonstration with conceptual spaces and subspace clustering,” in 2015 IEEE international conference on robotics and automation (ICRA) (Seattle, WA, USA: IEEE), 2592–2597. 10.1109/ICRA.2015.7139548

[B18] Deng Y. Wang M. Yang Y. Wang D. Yue Y. (2024). SEE-CSOM: sharp-edged and efficient continuous semantic occupancy mapping for mobile robots. IEEE Trans. Ind. Electron. 71, 1718–1728. 10.1109/TIE.2023.3262857

[B19] Du W. Ding C. Wu J. Xiong Z. (2024). Off-line programming framework for sorting task based on human-demonstration. IEEE Trans. Autom. Sci. Eng., 1–14. 10.1109/TASE.2024.3376712

[B20] Eiband T. Liebl J. Willibald C. Lee D. (2023). Online task segmentation by merging symbolic and data-driven skill recognition during kinesthetic teaching. Robot. Auton. Syst. 162, 104367. 10.1016/j.robot.2023.104367

[B21] Fitzgerald T. Goel A. Thomaz A. (2018). Human-guided object mapping for task transfer. ACM Trans. Hum.-Robot Interact. 7, 1–24. 10.1145/3277905

[B22] Fong J. Rouhani H. Tavakoli M. (2019). A therapist-taught robotic System for assistance during gait therapy targeting foot drop. IEEE Robot. Autom. Lett. 4, 407–413. 10.1109/LRA.2018.2890674

[B23] Fonooni B. Hellström T. Janlert L.-E. (2012). “Learning high-level behaviors from demonstration through semantic networks,” in 4th international conference on agents and artificial intelligence (ICAART), 419–426.

[B24] Fonooni B. Hellström T. Janlert L.-E. (2016). Priming as a means to reduce ambiguity in learning from demonstration. Int. J. Soc. Robot. 8, 5–19. 10.1007/s12369-015-0292-0

[B25] Fonooni B. Jevtić A. Hellström T. Janlert L.-E. (2015). Applying Ant Colony Optimization algorithms for high-level behavior learning and reproduction from demonstrations. Robot. Auton. Syst. 65, 24–39. 10.1016/j.robot.2014.12.001

[B26] French K. D. Kim J. H. Du Y. Goeddel E. M. Zeng Z. Jenkins O. C. (2023). Super intendo: semantic robot programming from multiple demonstrations for taskable robots. Robot. Auton. Syst. 166, 104397. 10.1016/j.robot.2023.104397

[B27] Guo Y. Xie Y. Chen Y. Ban X. Sadoun B. Obaidat M. S. (2022). An efficient object navigation strategy for mobile robots based on semantic information. Electronics 11, 1136. 10.3390/electronics11071136

[B28] Gustavsson O. Iovino M. Styrud J. Smith C. (2022). “Combining context awareness and planning to learn behavior trees from demonstration,” in 2022 31st IEEE international conference on robot and human interactive communication (RO-MAN) (Napoli, Italy: IEEE), 1153–1160. 10.1109/RO-MAN53752.2022.9900603

[B29] Hristov Y. Ramamoorthy S. (2021). “Learning from demonstration with weakly supervised disentanglement,” in 9th international Conference on learning representations, ICLR.

[B30] Hussein A. Gaber M. M. Elyan E. Jayne C. (2018). Imitation learning: a survey of learning methods. ACM Comput. Surv. 50, 1–35. 10.1145/3054912

[B31] Jankowski J. Racca M. Calinon S. (2022). From key positions to optimal basis functions for probabilistic adaptive control. IEEE Robot. Autom. Lett. 7, 3242–3249. 10.1109/LRA.2022.3146614

[B32] Kaelbling L. P. Oates T. Hernandez N. Finney S. (2001). “Learning in worlds with objects,” in Working notes of the AAAI stanford spring symposium on learning grounded representations, 31–36.

[B33] Kartmann R. Liu D. Asfour T. (2021). “Semantic scene manipulation based on 3D spatial object relations and language instructions,” in 2020 IEEE-RAS 20th international conference on humanoid robots (humanoids) (Munich, Germany: IEEE), 306–313. 10.1109/HUMANOIDS47582.2021.9555802

[B34] Kawasaki Y. Takahashi M. (2021). Bottom-up action modeling via spatial factorization for serving food. Adv. Robot. 35, 1–15. 10.1080/01691864.2021.1919548

[B35] Kollmitz M. Koller T. Boedecker J. Burgard W. (2020). “Learning human-aware robot navigation from physical interaction via inverse reinforcement learning,” in 2020 IEEE/RSJ international conference on intelligent robots and systems (IROS) (Las Vegas, NV, USA: IEEE), 11025–11031. 10.1109/IROS45743.2020.9340865

[B36] Kretzschmar H. Spies M. Sprunk C. Burgard W. (2016). Socially compliant mobile robot navigation via inverse reinforcement learning. Int. J. Robot. Res. 35, 1289–1307. 10.1177/0278364915619772

[B37] Kurenkov A. Akgun B. Thomaz A. L. (2015). “An evaluation of GUI and kinesthetic teaching methods for constrained-keyframe skills,” in 2015 IEEE/RSJ international conference on intelligent robots and systems (IROS) (Hamburg, Germany: IEEE), 3608–3613. 10.1109/IROS.2015.7353881

[B38] Kwak J. H. Lee J. Whang J. J. Jo S. (2022). Semantic grasping via a knowledge graph of robotic manipulation: a graph representation learning approach. IEEE Robot. Autom. Lett. 7, 9397–9404. 10.1109/LRA.2022.3191194

[B39] Lioutikov R. Neumann G. Maeda G. Peters J. (2015). “Probabilistic segmentation applied to an assembly task,” in 2015 IEEE-RAS 15th international conference on humanoid robots (humanoids) (Seoul, South Korea: IEEE), 533–540. 10.1109/HUMANOIDS.2015.7363584

[B40] Lourenço I. Bobu A. Rojas C. R. Wahlberg B. (2023). “Diagnosing and repairing feature representations under distribution shifts,” in 2023 62nd IEEE conference on decision and control (CDC) (Singapore, Singapore: IEEE), 3638–3645. 10.1109/CDC49753.2023.10383644

[B41] Markley F. L. Cheng Y. Crassidis J. L. Oshman Y. (2007). Averaging quaternions. J. Guid. Control Dyn. 30, 1193–1197. 10.2514/1.28949

[B42] Moon J. Lee B.-H. (2020). “Object-oriented semantic graph based natural question generation,” in 2020 IEEE international Conference on Robotics and automation (ICRA) (Paris, France: IEEE), 4892–4898. 10.1109/ICRA40945.2020.9196563

[B43] Muhlig M. Gienger M. Hellbach S. Steil J. J. Goerick C. (2009). “Task-level imitation learning using variance-based movement optimization,” in 2009 IEEE international conference on robotics and automation (Kobe: IEEE), 1177–1184. 10.1109/ROBOT.2009.5152439

[B44] Niekum S. Osentoski S. Konidaris G. Chitta S. Marthi B. Barto A. G. (2015). Learning grounded finite-state representations from unstructured demonstrations. Int. J. Robot. Res. 34, 131–157. 10.1177/0278364914554471

[B45] Pan Z. Polden J. Larkin N. Van Duin S. Norrish J. (2012). Recent progress on programming methods for industrial robots. Robot. Comput.-Integr. Manuf. 28, 87–94. 10.1016/j.rcim.2011.08.004

[B46] Pareek S. Kesavadas T. (2020). iART: learning from demonstration for assisted robotic therapy using LSTM. IEEE Robot. Autom. Lett. 5, 477–484. 10.1109/LRA.2019.2961845

[B47] Park F. C. Martin B. J. (1994). Robot sensor calibration: solving AX=XB on the Euclidean group. IEEE Trans. Robot. Autom. 10, 717–721. 10.1109/70.326576

[B48] Perez-D’Arpino C. Shah J. A. (2017). “C-LEARN: learning geometric constraints from demonstrations for multi-step manipulation in shared autonomy,” in 2017 IEEE international conference on robotics and automation (ICRA) (Singapore, Singapore: IEEE), 4058–4065. 10.1109/ICRA.2017.7989466

[B49] Peter G. (2000). Conceptual spaces: the geometry of thought. Cambridge, MA: MIT Press.

[B50] Qi X. Wang W. Yuan M. Wang Y. Li M. Xue L. (2020). Building semantic grid maps for domestic robot navigation. Int. J. Adv. Robot. Syst. 17, 172988141990006. 10.1177/1729881419900066

[B51] Ramirez-Amaro K. Dean-Leon E. Bergner F. Cheng G. (2019). A semantic-based method for teaching industrial robots new tasks. KI - Künstl. Intell. 33, 117–122. 10.1007/s13218-019-00582-5

[B52] Ravichandar H. Polydoros A. S. Chernova S. Billard A. (2020). Recent advances in robot learning from demonstration. Annu. Rev. Control Robot. Auton. Syst. 3, 297–330. 10.1146/annurev-control-100819-063206

[B53] Shek A. Su B. Y. Chen R. Liu C. (2023). “Learning from physical human feedback: an object-centric one-shot adaptation method,” in 2023 IEEE international Conference on Robotics and automation (ICRA) (London, United Kingdom: IEEE), 9910–9916. 10.1109/ICRA48891.2023.10161416

[B54] Steinmetz F. Nitsch V. Stulp F. (2019). Intuitive task-level programming by demonstration through semantic skill recognition. IEEE Robot. Autom. Lett. 4, 3742–3749. 10.1109/LRA.2019.2928782

[B55] Sun S. Zhao X. Li Q. Tan M. (2020). Inverse reinforcement learning-based time-dependent A* planner for human-aware robot navigation with local vision. Adv. Robot. 34, 888–901. 10.1080/01691864.2020.1753569

[B56] Villani V. Pini F. Leali F. Secchi C. Fantuzzi C. (2018). Survey on human-robot interaction for robot programming in industrial applications. IFAC-Pap. 51, 66–71. 10.1016/j.ifacol.2018.08.236

[B57] Ye G. Alterovitz R. (2017). “Demonstration-guided motion planning,” in Robotics research. Editors Christensen H. I. Khatib O. (Cham: Springer International Publishing), 291–307. 10.1007/978-3-319-29363-9_17

[B58] Zanchettin A. M. (2023). Symbolic representation of what robots are taught in one demonstration. Robot. Auton. Syst. 166, 104452. 10.1016/j.robot.2023.104452

[B59] Zhang Y. Tian G. Shao X. Zhang M. Liu S. (2023). Semantic grounding for long-term autonomy of mobile robots toward dynamic object search in home environments. IEEE Trans. Ind. Electron. 70, 1655–1665. 10.1109/TIE.2022.3159913

[B60] Ziaeetabar F. Aksoy E. E. Worgotter F. Tamosiunaite M. (2017). “Semantic analysis of manipulation actions using spatial relations,” in 2017 IEEE international conference on robotics and automation (ICRA) (Singapore, Singapore: IEEE), 4612–4619. 10.1109/ICRA.2017.7989536

