# Supplementary material for: Semantic learning from keyframe demonstration using object attribute constraints
Source: Front Robot AI. 2024 Jul 18;11:1340334. doi: 10.3389/frobt.2024.1340334 (PMC11292798; doi:10.3389/frobt.2024.1340334)
Supplement: Supplementary file 2 [file DataSheet1.PDF]

Supplementary Material

Semantic Learning from Keyframe Demonstration using Object Attribute Constraints

Busra Sen*, Jos Elfring, Elena Torta, and René van de Molengraft

*** Correspondence:**
Busra Sen
b.sen@tue.nl

Algorithm SI: Reproduction Stage

**Input**	:	Constraints, The number of reference Objects, Object Attributes and Object Poses in Scene
**Output**	:	Desired End-effector Poses
Step 1	→	**Check constraints for each keyframe group**

Initialize required keyframe clusters with candidate objects in reproduction

m-1: the number of reference objects' group, ***q:*** the number of situational objects

Find the candidate objects for keyframe group c_i , i = {2, 3, …,m+q-1}

Initialize Candidate Object Vectors →

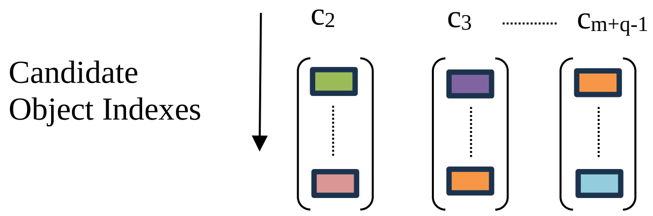

Candidate Object Matrix →

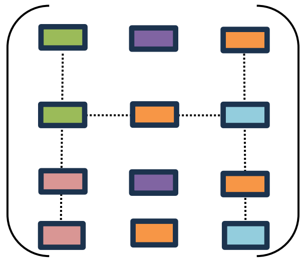

Remove rows, if any two columns have the same object indexes

Step 2	→	**If they exist, apply spatial Relation constraints between reference objects and situational objects**<br>Take two relevant columns from the candidate object matrix.<br>Compute Spatial Relations for each object pair:<br>$(C_a,\ C_b), \forall\{a, b\} \in [2, m+q-1]$<br>If spatial relation is not equal to the spatial constraint label:<br>Remove the corresponding rows<br>Update Candidate Object Matrix
Step 3	→	**If they exist, apply inter-object discrete and continuous constraints between each keyframe group**<br>Take relevant columns from the candidate object matrix.<br>If the constraint is discrete : Remove the corresponding rows if the attribute to be constrained is not equal for candidate objects.<br>If the constraint is continuous : Remove the corresponding rows if the attribute to be constrained is not valid for the linear equation.
Step 4	→	**If the row number of the result of the candidate object matrix == 1:**<br><br>Actual Reference Object Poses → The poses in the first m-1 column of the matrix<br>Use the desired relative poses between the end-effector and the object, and actual reference object poses : Compute the desired end-effector poses

Table S1 The result of candidate objects for each keyframe in reproduction for the stacking task

	Candidate Objects after Constraints Between Demonstrations	Candidate Objects after Discrete Inter-Object Constraints	Result
**Case 1:** O_1-O_7	c_2 : $\{O_1\}$<br>c_3 : $\{O_7\}$	c_2 : None<br>c_3 : None	Fail
**Case 2:** O_1-O_4-O_7-O_8	c_2: $\{O_1, O_4\}$<br>c_3: $\{O_7, O_8\}$	c_2: $\{O_4\}$<br>c_3: $\{O_8\}$	Success
**Case 3:** O2-O6-O9	c_2: $\{O_2\}$<br>c_3: $\{O_6\}$	c_2: $\{O_2\}$<br>c_3: $\{O_6\}$	Success

Table S2 The result of candidate objects for each keyframe in reproduction for the sorting task

Reproduction Scenes	Candidate Objects after Constraints Between Demonstrations	Candidate Objects after Inter-Object Discrete Constraints	Candidate Objects after Inter-Object Continuous Constraints

**Case 1:** O_{10}-O_{11}-O_{12}-O_6 -O_7-O_{13}	**c_2**:$\{O_{10},O_{11},O_{12},O_6,O_7\}$<br>**$c_3$**:$\{O_{13}\}$<br>**$c_4$**:$\{O_{10},O_{11},O_{12},O_6,O_7\}$<br>**$c_5$**:$\{O_{13}\}$<br>**$c_6$**:$\{O_{11},O_{12},O_7\}$<br>**$c_7$**:$\{O_{13}\}$	**c_2** : $\{O_{10}, O_{11},O_{12}\}$<br>**$c_3$** : $\{O_{13}\}$<br>**c_4** : $\{O_{10}, O_{11},O_{12}\}$<br>**$c_5$** : $\{O_{13}\}$<br>**c_6** : $\{O_{11},O_{12}\}$<br>**c_7** : $\{O_{13}\}$	**c_2** : $\{O_{10}\}$<br>**c_3** : $\{O_{13}\}$<br>**c_4** : $\{O_{11}\}$<br>**c_5** : $\{O_{13}\}$<br>**c_6** : $\{O_{12}\}$<br>**c_7** : $\{O_{13}\}$
**Case 2:** O_1 – O_3- O_7-O_6-O_5-O_8 -O_{13}	**c_2**:$\{O_1,O_3,O_7,O_6,O_8\}$<br>**c_3**:$\{O_{13}\}$<br>**c_4**:$\{O_1,O_3,O_7,O_6,O_5,O_8\}$<br>**$c_5$**:$\{O_{13}\}$<br>**$c_6$**:$\{O_3,O_7, O_5,O_8\}$<br>**$c_7$**:$\{O_{13}\}$	**c_2**: $\{O_1,O_3,O_7,O_6,O_8\}$<br>**c_3**:$\{O_{13}\}$<br>**c_4**: $\{O_1,O_3,O_7,O_6,O_5,O_8\}$<br>**$c_5$**:$\{O_{13}\}$<br>**$c_6$**: $\{O_3,O_7, O_5,O_8\}$<br>**c_7**:$\{O_{13}\}$	**c_2** : $\{O_6\}$<br>**c_3** : $\{O_{13}\}$<br>**c_4** : $\{O_7\}$<br>**c_5** : $\{O_{13}\}$<br>**c_6** : $\{O_8\}$<br>**c_7** : $\{O_{13}\}$
**Case 3:** O_7 – O_{10}- O_8-O_{12}-O_{11}-O_1 -O_{13}	**c_2**:$\{O_7,O_{10},O_8,O_{12},O_{11},O_1\}$<br>**$c_3$**:$\{O_{13}\}$<br>**$c_4$**: $\{O_7,O_{10},O_8,O_{12},O_{11},O_1\}$<br>**$c_5$**:$\{O_{13}\}$<br>**$c_6$**:$\{O_7, O_8,O_{12},O_{11}\}$<br>**$c_7$**:$\{O_{13}\}$	**c_2**:$\{O_{10},O_{11},O_{12}\}$<br>**c_3**:$\{O_{13}\}$<br>**c_4**:$\{O_{10},O_{11},O_{12}\}$<br>**c_5**:$\{O_{13}\}$<br>**c_6**:$\{O_{11},O_{12}\}$<br>**c_7**:$\{O_{13}\}$	**c_2** : $\{O_{10}\}$<br>**c_3** : $\{O_{13}\}$<br>**c_4** : $\{O_{11}\}$<br>**c_5**: $\{O_{13}\}$<br>**c_6**: $\{O_{12}\}$<br>**c_7**: $\{O_{13}\}$

Table S3 The result of candidate objects for each keyframe in reproduction for the serving task

Reproduction Scenes	***Candidate Objects after Constraints Between Demonstrations***	***Candidate Objects after Spatial Constraints***	***Candidate Objects after Inter-task Discrete Constraints***
Case 1: O_4- O_3- O_{10}- O_6- O_7	**c_2** : $\{O_4, O_3, O_{10}\}$<br>**c_3** : $\{O_6\}$<br>**c_4** : $\{O_7\}$<br>**c_5**: $\{O_4, O_3, O_{10}\}$<br>**c_6** : $\{O_4 ,O_3, O_{10}\}$	**c_2** : $\{O_3, O_4\}$<br>**c_3**: $\{O_6\}$<br>**c_4** : $\{O_7\}$<br>**c_5**: $\{O_3, O_4\}$<br>**c_6** : $\{O_{10}\}$	**c_2** : $\{O_4\}$<br>**c_3** : $\{O_6\}$<br>**c_4** : $\{O_7\}$<br>**c_5** : $\{O_3\}$<br>**c_6** : $\{O_{10}\}$

Case 2: O_1- O_2- O_7- O_9- O_4-O_{14}- O_{11}-O_6	**c_2** : {O_1, O_2, O_4, O_9, O_{11}}	**c_2** : {O_1, O_2, O_4, O_9}	**c_2** : {O_9}
	**c_3** : {O_6}	**c_3** : {O_6}	**c_3** : {O_6}
	**c_4** : {O_7, O_{14}}	**c_4** : {O_7, O_{14}}	**c_4** : {O_{14}}
	**c_5** : {O_1, O_2, O_4, O_9, O_{11}}	**c_5** : {O_1, O_2, O_4, O_9}	**c_5** : {O_4}
	**c_6** : {O_1, O_2, O_4, O_9, O_{11}}	**c_6** : {O_{11}}	**c_6** : {O_{11}}
Case 3: O_4- O_6- O_{12}- O_{13}- O_7- O_5-O_{11}	**c_2** : {O_5, O_{11},O_{12}, O_{13}, O_4}	**c_2** : {O_{13}}	**c_2** : {O_{13}}
	**c_3** : {O_6}	**c_3** : {O_6}	**c_3** : {O_6}
	**c_4** : {O_7}	**c_4** : {O_7}	**c_4** : {O_7}
	**c_5** : { O_{11},O_{12}, O_4}	**c_5** : {O_{12}}	**c_5** : {O_{12}}
	**c_6** : { O_{11},O_{12}, O_4}	**c_6** : {O_4}	**c_6** : {O_4}
